# Morphological and Optical Properties of RE-Doped ZnO Thin Films Fabricated Using Nanostructured Microclusters Grown by Electrospinning–Calcination

**DOI:** 10.3390/nano15171369

**Published:** 2025-09-04

**Authors:** Marina Manica, Mirela Petruta Suchea, Dumitru Manica, Petronela Pascariu, Oana Brincoveanu, Cosmin Romanitan, Cristina Pachiu, Adrian Dinescu, Raluca Muller, Stefan Antohe, Daniel Marcel Manoli, Emmanuel Koudoumas

**Affiliations:** 1 National Institute for Research and Development in Microtechnologies—IMT Bucharest, 126A, Erou Iancu Nicolae Street, 077190 Voluntari-Bucharest, Romania; marina.manica@imt.ro (M.M.); dumitru.manica@imt.ro (D.M.); oana.brincoveanu@imt.ro (O.B.); cosmin.romanitan@imt.ro (C.R.); cristina.pachiu@imt.ro (C.P.); adrian.dinescu@imt.ro (A.D.); raluca.muller@imt.ro (R.M.); 2 R&D Center for Materials and Electronic & Optoelectronic Devices (MDEO), Faculty of Physics, University of Bucharest, Atomiștilor Street 405, 077125 Măgurele, Ilfov, Romania; 3 Center of Materials Technology and Photonics (CEMATEP), School of Engineering and Research and Innovation Center (PEK), Hellenic Mediterranean University (HMU), 71410 Heraklion, Crete, Greece; 4 “Petru Poni” Institute of Macromolecular Chemistry, 41A Grigore Ghica Voda Alley, 700487 Iasi, Romania; 5 Academy of Romanian Scientists (AOSR), Ilfov Street 3, 050045 Bucharest, Romania; 6 Soil Mechanics and Foundation Engineering, Technical University of Civil Engineering Bucharest, 020396 Bucharest, Romania

**Keywords:** ZnO thin films, rare-earth doping, electrospinning–calcination, nanostructured microclusters, La:ZnO, Er:ZnO, Sm:ZnO, bandgap tuning, Raman spectroscopy, transparent optoelectronics

## Abstract

In this study, we report the fabrication and multi-technique characterization of pure and rare-earth (RE)-doped ZnO thin films using nanostructured microclusters synthesized via electrospinning followed by calcination. Lanthanum (La), erbium (Er), and samarium (Sm) were each incorporated at five concentrations (0.1–5 at.%) into ZnO, and the resulting powders were drop-cast as thin films on glass substrates. This approach enables the transfer of pre-engineered nanoscale morphologies into the final thin-film architecture. The morphological analysis by scanning electron microscopy (SEM) revealed a predominance of spherical nanoparticles and nanorods, with distinct variations in size and aspect ratio depending on dopant type and concentration. X-ray diffraction (XRD) and Rietveld analysis confirmed the wurtzite ZnO structure with increasing evidence of secondary phase formation at high dopant levels (e.g., Er_2_O_3_, Sm_2_O_3_, and La(OH)_3_). Raman spectroscopy showed peak shifts, broadening, and defect-related vibrational modes induced by RE incorporation, in agreement with the lattice strain and crystallinity variations observed in XRD. Elemental mapping (EDX) confirmed uniform dopant distribution. Optical transmittance exceeded 70% for all films, with Tauc analysis revealing slight bandgap narrowing (Eg = 2.93–2.97 eV) compared to pure ZnO. This study demonstrates that rare-earth doping via electrospun nanocluster precursors is a viable route to engineer ZnO thin films with tunable structural and optical properties. Despite current limitations in film-substrate adhesion, the method offers a promising pathway for future transparent optoelectronic, sensing, or UV detection applications, where further interface engineering could unlock their full potential.

## 1. Introduction

Zinc oxide (ZnO) is a wide-bandgap semiconductor (Eg ~3.3 eV) with a hexagonal wurtzite structure, attracting growing attention for its multifunctionality in optoelectronic, sensing, and transparent device applications [1,2]. Its inherent advantages—such as high exciton binding energy, chemical stability, and abundant availability—have prompted extensive research into tailoring its properties via doping and nanostructuring [3]. Among the dopants explored, rare-earth elements (REs) such as lanthanum (La), erbium (Er), and samarium (Sm) offer distinctive opportunities for property modulation, owing to their large ionic radii, multiple oxidation states, and capability to induce localized states within the ZnO band structure [4,5,6].

Recent advances in bottom-up synthesis techniques, including electrospinning, have enabled the preparation of ZnO-based nanostructures with controlled morphology and enhanced defect chemistry [7,8,9,10,11,12,13,14,15,16,17,18,19]. However, few studies have investigated the integration of such nanostructured precursors into thin films for functional applications. In this work, we explore a hybrid synthesis route in which RE-doped ZnO nanostructured microclusters—obtained by electrospinning followed by calcination [12]—are dispersed in isopropanol and deposited as thin films via drop-casting. This method offers the advantage of transferring pre-defined nanoscale architectures and doping profiles directly into film form, potentially overcoming limitations associated with in situ doping during vapor-phase deposition [20]. Transferring pre-defined nanoscale architectures and doping profiles directly into film form offers several advantages, particularly in the fields of electronics, photonics, and materials science. This approach allows for precise control over the material properties and functionalities at the nanoscale, which is crucial for the development of advanced devices. By integrating these architectures and doping profiles into films, it is possible to enhance device performance, scalability, and versatility. Some specific advantages of this approach are underline by recent publications such as the following. Doping ZnO with rare-earth elements can significantly enhance its optical properties, such as photoluminescence, by facilitating efficient energy transfer from the ZnO host to the rare-earth ions. This is particularly beneficial for applications in optoelectronics and photonics, where improved luminescence is desired [4,6]. The incorporation of rare-earth elements like lanthanum into ZnO films can lead to a reduction in the bandgap, thereby improving the material’s electrical conductivity and making it more suitable for applications in dye-sensitized solar cells [21]. Doped ZnO films exhibit improved thermoluminescence properties, which are advantageous for radiation dosimetry applications. The presence of rare-earth elements enhances the material’s sensitivity and stability under radiation exposure [22]. The ability to maintain the hexagonal wurtzite structure of ZnO even after doping with rare-earth elements ensures that the material retains its desirable mechanical and chemical stability, which is crucial for long-term device performance [9,21,23]. Doped ZnO films can be tailored for a wide range of applications, including transparent conducting oxides for flexible electronics, gas sensors, and bio-imaging. The versatility of ZnO, combined with the tunable properties introduced by doping, makes it a highly attractive material for various technological applications [4,14,15]. The direct transfer of nanoscale architectures into film form allows for the integration of ZnO-based materials into existing device architectures, facilitating the development of advanced optoelectronic devices with enhanced performance. While the advantages of transferring doped ZnO nanoscale architectures into film form are significant, it is important to consider potential challenges. For instance, achieving uniform doping and maintaining the desired structural properties across large areas can be technically challenging. Additionally, the choice of dopants and their concentrations must be carefully optimized to avoid adverse effects on the material’s properties. Despite these challenges, the potential benefits of this approach make it a promising way for advancing the performance of ZnO-based materials in various applications. The added value of this work lies in the unique combination of material design and processing route. The rare-earth (RE)-doped ZnO films reported here are obtained from nanostructured microclusters synthesized via electrospinning followed by calcination, a morphology–composition configuration that, to the best of our knowledge, has not been reported before in thin film form. The present study focuses on translating these pre-formed nanostructured powders—previously optimized for structural and functional properties—into films/coatings while preserving their intrinsic 3D microcluster architecture and associated properties. This powder-to-film transfer is non-trivial, as most studies on RE-doped ZnO films rely on direct in situ growth methods that result in different morphologies and property sets. Our approach opens opportunities for optoelectronic, photocatalytic, and EMI shielding applications where such high-surface-area, porous, yet transparent architectures are beneficial. We investigate the influence of RE dopant type (La, Er, and Sm) and concentration (0.1–5 at.%) on the morphology, structural parameters, vibrational modes, and optical bandgap of ZnO thin films. Detailed characterization using SEM, EDX, XRD with Rietveld refinement, Raman spectroscopy, and UV-Vis spectroscopy provides insight into dopant-induced modifications and phase evolution. The findings highlight key structure–property correlations and suggest promising applications in UV photodetectors, transparent sensors, and future flexible electronics, pending optimization of film–substrate adhesion through optically inert interfacial layers.

## 2. Materials and Methods

### 2.1. Synthesis of RE-Doped ZnO Nanostructured Microclusters

Nanostructured microclusters of pure ZnO and RE-doped ZnO (La:ZnO, Er:ZnO, and Sm:ZnO) were synthesized via electrospinning followed by thermal treatment [12]. Precursor solutions were prepared by dissolving zinc acetate dihydrate in a polyvinylpyrrolidone (PVP)–ethanol system, followed by the addition of calculated amounts of La(NO_3_)_3_·6H_2_O, Er(NO_3_)_3_·5H_2_O, or Sm(NO_3_)_3_·6H_2_O to yield target dopant concentrations of 0.1, 0.5, 1, 2.5, and 5 at.% RE relative to Zn^2+^. All the precursor materials and solvents were provided by Sigma Aldrich (St. Louis, MO, USA) with analytical-grade purity (≥99%). The mixtures were stirred until complete homogenization and loaded into a syringe equipped with a stainless-steel needle for electrospinning.

The electrospinning process was carried out at an applied voltage of 20 kV, a feed rate of 3 mL/h, and a tip-to-collector distance of 15 cm. A rotating drum collector was used to ensure uniform deposition of the nanofibers. The as-spun mats were subsequently calcined at 700 °C for 2 h in air to obtain RE:ZnO nanostructured microclusters composed of aggregated nanocrystals and nanorods.

### 2.2. Preparation of Thin Films by Drop-Casting

To fabricate thin films, 5 mg of each RE:ZnO nanostructured powder was dispersed in 5 mL of isopropanol and subjected to ultrasonication for 30 min to ensure homogeneous suspension. A fixed volume of the resulting dispersion was drop-cast (21 drops) onto cleaned 2 × 2 cm glass substrates and allowed to dry at ambient conditions. This procedure produced continuous but weakly adherent thin films due to the absence of binding agents or sintering. Future improvements are anticipated through the use of optically inactive adhesion promoters.

### 2.3. Characterization Techniques

The morphology of the thin films was examined using scanning electron microscopy (SEM, Nova NanoSEM 630, FEI, Hillsboro, OR, USA) operated at 10 kV acceleration voltage and working distance from 5 to 6 mm according to the samples needs. Particle and nanorod size distributions were extracted using ImageJ v1.54p software (National Institutes of Health (NIH), Bethesda, MD, USA) from at least 200 features per sample. Elemental composition and dopant distribution were investigated by energy-dispersive X-ray spectroscopy (EDX) and compositional mapping. Structural properties were assessed via X-ray diffraction (XRD, grazing incidence mode at 0.5°, 40 kV, 75 mA) using a SmartLab, (Rigaku Corporation, Tokyo, Japan) diffractometer. Rietveld refinement was applied to determine average crystallite size and lattice strain. The Rietveld refinement was performed using the Whole Powder Pattern Fitting (Rietveld) method integrated in PDXL: Integrated X-ray powder diffraction commercial software developed by Rigaku corporation. Raman spectroscopy was performed using a WITec Alpha300 R (WITec GmbH, Ulm, Germany) microscope with a 532 nm laser to evaluate phonon modes, lattice disorder, and defect states. UV-Vis optical transmittance and absorbance spectra were recorded in the 300–900 nm range using a Ossila (Ossila Ltd., Sheffield, UK) spectrophotometer. The optical bandgap (Eg) was calculated from Tauc plots assuming a direct allowed transition.

## 3. Results and Discussion

### 3.1. Morphological Analysis of RE:ZnO Thin Films

**Figure 1:**
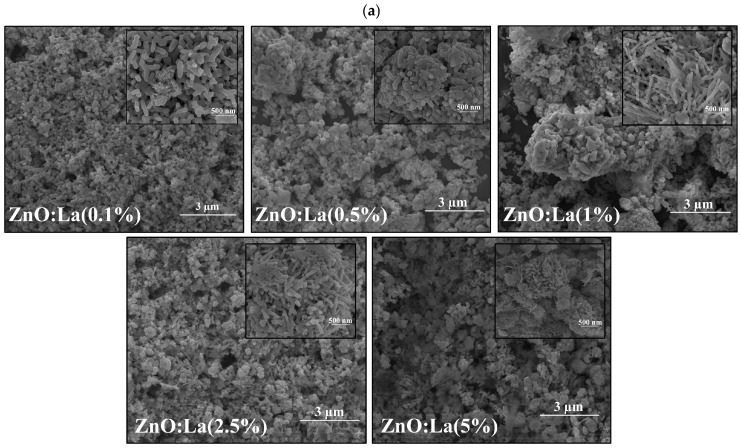

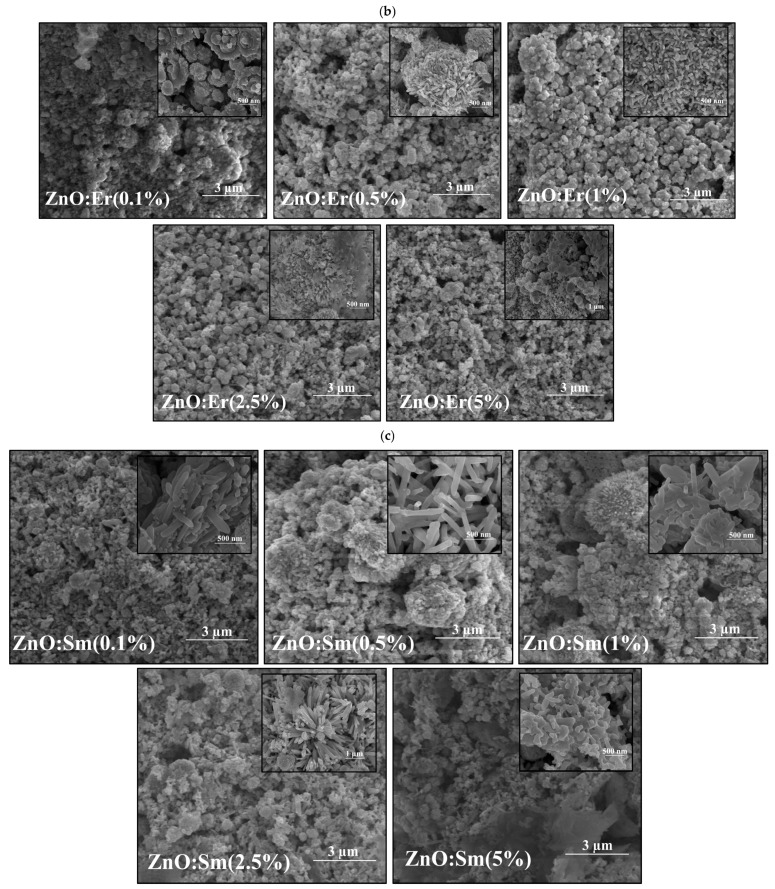
SEM images of different dopant concentrations for RE:ZnO thin films: (**a**) La:ZnO, (**b**) Er:ZnO, and (**c**) Sm:ZnO. Inset shows details of clusters’ nanostructure at ×100,000 magnification, scale 500 nm.

The surface morphology of pure and RE-doped ZnO (La:ZnO, Er:ZnO, Sm:ZnO) thin films was analyzed using SEM. Representative micrographs revealed nanostructured surfaces composed primarily of spherical nanoparticles and, in several cases, elongated nanorod-like features. The size, distribution, and relative abundance of these morphological features were strongly dependent on both the type and concentration of the RE dopant, as one can see in Figure 1a–c.

In the case of La:ZnO films, both spherical nanoparticles and nanorods were observed across all concentrations (0.1–5 at.%). As the La concentration increased, the average diameter of the nanoparticles exhibited a non-monotonic trend, peaking at 1 at.% (86.6 ± 21.1 nm) and slightly decreasing at higher concentrations. Meanwhile, the nanorods showed increased polydispersity, with average lengths ranging from 327.5 ± 81.8 nm (0.5% La) to 442.3 ± 237.5 nm (5% La). These trends suggest that La^3+^ incorporation promotes anisotropic growth at higher concentrations, potentially due to its large ionic radius (1.17 Å), which induces local strain and affects crystallite growth dynamics.

Er:ZnO thin films displayed a predominantly spherical morphology without significant rod-like structures, even at higher dopant levels. Nanoparticle size increased progressively with Er content, reaching an average diameter of 133.1 ± 52.5 nm at 5 at.%. This trend indicates a tendency toward particle coalescence or aggregation with increasing Er^3+^ content, likely due to the dopant’s ability to induce localized strain fields that favor grain growth.

Sm:ZnO films exhibited the most complex morphology, with both nanospheres and high-aspect-ratio nanorods present at all concentrations. At 2.5 at.% Sm, nanorods with an average length exceeding 600 nm and widths around 50 nm were observed, reaching up to ~940 nm in length at 5 at.% Sm. These results suggest a dopant-induced directional growth mechanism possibly enhanced by Sm^3+^ acting as a morphological promoter under thermal processing. The formation of elongated nanostructures at high Sm levels is of particular interest for anisotropic applications such as photodetectors, etc. ***More information about particle size evaluation is included in *Appendix A**, Table A1, Table A2, Table A3, Table A4, Table A5 and Table A6.

In all cases, the films appeared continuous at the microscale, but a modest adhesion to the glass substrate was noted, attributed to the absence of sintering agents or interfacial layers. Future work will focus on introducing optically inert additives to improve adhesion without compromising transparency or functionality.

Overall, the morphology of RE:ZnO films is tunable by both dopant selection and concentration. The trends suggest that La promotes nanorod growth, Er favors particle coarsening, and Sm induces significant anisotropy in both particle and rod formation, providing a versatile platform for tailoring surface structure for targeted optoelectronic applications.

### 3.2. Structural Analysis: XRD and Rietveld Refinement

**Figure 2:**
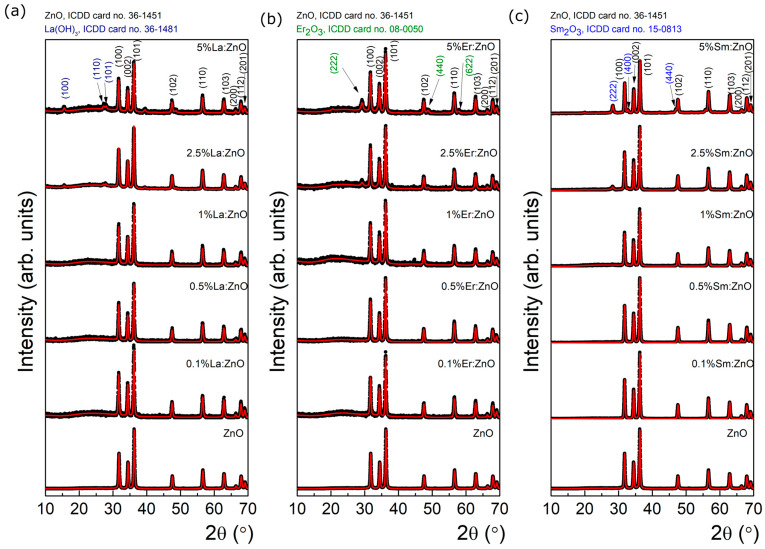
XRD characterization of different dopant concentrations for RE:ZnO thin films: (**a**) La:ZnO, (**b**) Er:ZnO, and (**c**) Sm:ZnO.

X-ray diffraction (XRD) analysis in grazing incidence geometry (0.5°) was employed to assess the crystal structure and phase composition of the RE:ZnO thin films. All samples primarily exhibited the hexagonal wurtzite ZnO phase (JCPDS No. 36-1451), characterized by strong reflections corresponding to the (100), (002), and (101) planes, confirming the preservation of the ZnO crystalline lattice after drop-casting of electrospun–calcined powders, as observed from Figure 2. In order to clarify the successful transfer of the powders to films/coatings, XRDs of initial powders were added to the manuscript in Appendix A, Table A10. The XRDs were recorded in powder form (blue line) and final material as a film on glass substrate (black line). One can note the presence of specific diffraction peaks of wurtzite ZnO before and after drop casting, without any shift. For Sm and La, after drop-casting, no additional diffraction peaks can be observed. For Er-doped ZnO thin films at 1% and 2.5%, we observe two very small diffraction peaks, at ~44° and ~39°, respectively, present only in the films, which could be ascribed to accidental metal contamination possibly from the glass substrates consisting of common microscope slides glass.

As it can be observed from the XRD spectra, in La:ZnO films, the diffraction peaks remained sharp and well-defined for low La concentrations (≤1 at.%), suggesting good crystallinity. However, at 2.5 and 5 at.% La, additional peaks were observed, which matched the orthorhombic La(OH)_3_ phase (JCPDS No. 36-1481), indicating the formation of secondary phases at high dopant loadings. This can be attributed to the limited solubility of La^3+^ in ZnO and its tendency to segregate at grain boundaries when its concentration exceeds the substitutional capacity of the host lattice. For Er:ZnO, the XRD patterns revealed pure ZnO signatures at 0.1 and 0.5 at.% Er, while higher dopant levels led to the emergence of weak peaks at 2θ ≈ 29.2°, 48.9°, and 58.1°, corresponding to the (222), (400), and (622) planes of cubic Er_2_O_3_ (JCPDS No. 08-0050). This indicates that Er^3+^ incorporation is possible up to a certain threshold, beyond which phase segregation occurs. Similarly, Sm:ZnO films displayed only ZnO peaks up to 1 at.% Sm, whereas small additional reflections appeared at 2.5 and 5 at.% dopant concentration. These were attributed to cubic Sm_2_O_3_ (JCPDS No. 15-9813), with main reflections at 2θ ≈ 27.7°, 32.8°, and 45.1°. The tendency for phase separation at higher Sm content further supports the limited solubility of RE ions in ZnO.

Rietveld refinement of the XRD data provided quantitative insight into crystallite size (τ) and microstrain (ε) evolution with doping (Table 1). Rietveld refinement is based on the least squared method of the theoretical profile against the experimental one in which each diffraction peak may be described by a symmetric, or nearly symmetric, pseudo-Voigt function. It requires the reasonable initial approximation of many free parameters, including peak shape, unit cell dimensions, or coordinates of all atoms in the crystal structure. In particular, such an analysis is important taking into account that the size of the crystalline domains (called mean crystallite size) and the lattice strain govern the structural defect formation at the boundaries of crystallites. More details were reported in some previous works [19].

In all RE:ZnO systems, a reduction in τ and an increase in ε were observed upon doping, consistent with substitutional stress and lattice distortion induced by RE^3+^ ions. For La:ZnO, τ slightly increased with dopant level up to 1 at.% (from 23.3 nm to 24.8 nm), followed by the emergence of La(OH)_3_ domains at higher levels, which had distinct crystallite sizes (~12–15 nm). In contrast, Er:ZnO and Sm:ZnO films exhibited a consistent reduction in crystallite size τ and simultaneous growth of secondary phases with very small crystallites (τ ≈ 8–10 nm), supporting the formation of strain-inducing inclusions.

These structural findings align with the Raman results (Section 3.3) and confirm that rare-earth doping influences ZnO lattice integrity in a concentration-dependent manner. Moderate doping up to 1% preserves the wurtzite phase and introduces beneficial strain, while larger doping concentrations (2.5, 5%) promote the formation of RE-oxide inclusions, which may impact charge transport, optical transparency, and phonon behavior.

These results confirm that the electrospun-derived RE:ZnO thin films maintain structural integrity up to moderate doping levels and that phase separation at higher RE content introduces new functionalities but may compromise crystallinity.

### 3.3. Raman Spectroscopy

**Figure 3:**
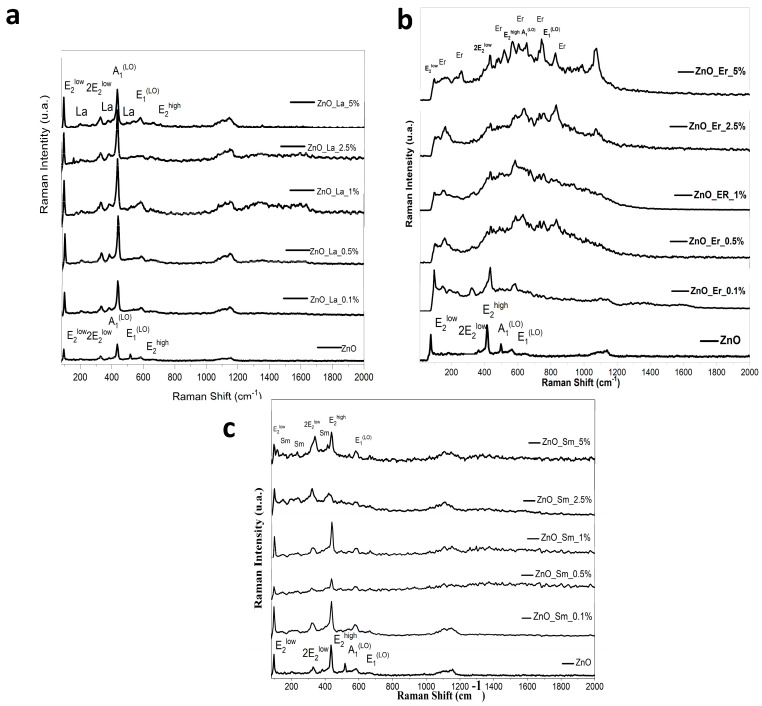
Raman spectra of different dopant concentrations for RE:ZnO thin films: (**a**) La:ZnO, (**b**) Er:ZnO, and (**c**) Sm:ZnO.

Raman spectroscopy was employed to evaluate the vibrational properties and lattice integrity of the RE:ZnO thin films. Raman spectra for all samples (Figure 3) revealed the characteristic modes of the wurtzite ZnO structure, with modifications induced by RE doping. The key modes identified include the E_2_ (low), E_2_ (high), A_1_(LO), and E_1_(LO) phonons, whose positions, intensities, and linewidths were sensitive to the dopant type and concentration.

In undoped ZnO, strong E_2_ (high) (~437 cm^−1^) and E_2_ (low) (~99 cm^−1^) peaks were observed, indicative of high crystalline quality and low defect density. The E_2_ (high) mode is particularly sensitive to lattice order and is considered a fingerprint of wurtzite ZnO [24].

**La:ZnO** films exhibited a progressive shift and broadening of the E_2_ (high) and E_1_(LO) (~583 cm^−1^) modes with increasing La content. At low concentrations (0.1–0.5 at.%), the E_2_ (high) mode intensified slightly, suggesting a modest increase in crystalline order. However, at higher La levels (≥1 at.%), the E_2_ (high) mode broadened and decreased in intensity, accompanied by the emergence of additional peaks around 391 cm^−1^ and above 1100 cm^−1^, which are attributed to local vibrational modes induced by La^3+^ or La-related secondary phases (La(OH)_3_). The increased intensity and broadening of A_1_(LO) and E_1_(LO) modes point to enhanced defect formation and carrier concentration, in agreement with XRD and UV-Vis results.

**Er:ZnO** samples displayed a distinct Raman signature, with new peaks appearing between 150 and 300 cm^−1^ and between 700 and 800 cm^−1^ as Er concentration increased. These bands are assigned to Er–O and Er–Zn vibrational modes or to Er_2_O_3_ nanodomains, corroborating the secondary phase formation observed in XRD. A systematic red-shift and significant broadening of the E_2_ (high) and E_1_(LO) modes were recorded as Er content increased from 0.1% to 5%, suggesting increased lattice strain and phonon–defect coupling. At 5 at.% Er, the E_2_ (high) mode was severely damped, consistent with reduced ZnO crystallinity and partial amorphization induced by dopant clustering or phase segregation.

**Sm:ZnO** spectra showed similar trends, with clear Raman features corresponding to ZnO preserved at low doping levels (0.1–1%) but increasingly distorted at 2.5–5 at.%. New peaks at ~490 cm^−1^ and ~678 cm^−1^ emerged, attributed to Sm_2_O_3_ vibrational modes. The E_2_ (high) mode became progressively weaker and broader, while E_1_(LO) and multifonon bands (~1100–1200 cm^−1^) intensified with Sm content, implying higher defect densities and potential oxygen vacancies or Zn interstitials. Notably, the presence of long nanorods in Sm:ZnO correlated with more pronounced Raman mode anisotropy, indicating the influence of morphology on phonon confinement effects.

These observayions are summarized in Table 2.

**Figure 4:**
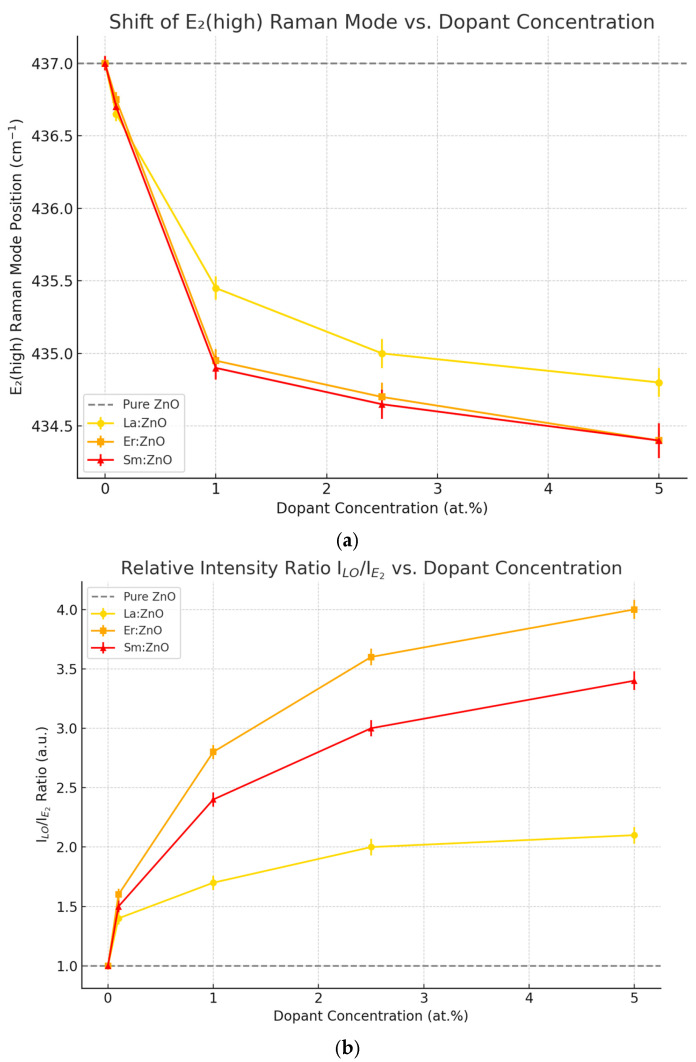
(**a**) E_2_ (high) Raman mode position vs. dopant concentration shows a progressive redshift (downshift in wavenumber) as RE dopant concentration increases. Er and Sm dopants induce stronger shifts compared to La, indicating more pronounced lattice distortion. (**b**) **I_LO_/I_E2_ intensity ratio vs. dopant concentration** illustrates increasing defect-related polar LO mode intensity relative to the E_2_ (high) phonon. Er doping causes the most significant rise, consistent with stronger defect generation and polar coupling.

The Raman results confirm that RE doping modifies the phonon landscape of ZnO through lattice distortion, defect generation, and local symmetry breaking. These effects are minimal at ≤1 at.% doping but become dominant at ≥2.5 at.%, where secondary phases are detected, as can be observed in Figure 4. Among the REs investigated, La introduces the most subtle modifications (until phase segregation), while Er and Sm induce stronger vibrational disorder and spectral complexity at comparable concentrations. These findings are consistent with the XRD (Section 3.2) and optical (Section 3.5) analyses, reinforcing the structure–property correlations governed by dopant nature, concentration, and ionic radius. For Sm:ZnO coatings, E_2_ (low) downshifts and LO modes strengthen with concentration, reflecting local mass/strain perturbations and anisotropy of the rod-rich surface. GI-XRD confirms retention of the wurtzite ZnO phase with only weak Sm_2_O_3_ reflections at ≥2.5 at.%, i.e., long-range crystallinity remains largely intact. The stronger Raman disorder signature therefore reflects local fields and defect–phonon coupling rather than a large loss of ZnO phase fraction.

### 3.4. Elemental Composition and Dopant Distribution (EDX Analysis)

The incorporation of rare-earth dopants into the ZnO matrix was confirmed through energy-dispersive X-ray spectroscopy (EDX) performed on selected RE:ZnO thin films. Elemental quantification and mapping were used to validate both the presence and the homogeneity of La, Er, and Sm within the films deposited on glass substrates and are presented in Table A7, Table A8 and Table A9 in Appendix A.

For all three RE systems, EDX spectra revealed clear peaks corresponding to Zn, O, and the respective dopant element (La, Er, or Sm). The detected atomic percentages closely matched the nominal doping levels (0.1–5 at.%), confirming the accuracy and reproducibility of the synthesis and deposition process. Importantly, compositional mapping images demonstrated a uniform spatial distribution of RE dopants across the film surface, with no significant clustering or phase separation observable at the micron scale.

**La:ZnO, Er:ZnO, and Sm:ZnO films** were analyzed in detail. Table 3 summarizes the elemental composition obtained from point analysis on the film surface. At low concentrations (0.1–1 at.%), Sm was successfully detected with atomic fractions between 0.6% and 1.1%, aligning well with target values. At 2.5% and 5% Sm, an increase in both absolute and relative Sm content was observed, accompanied by a slight decrease in Zn content, suggesting partial substitution or surface segregation. These trends are consistent with the XRD-detected emergence of Sm_2_O_3_ at higher doping levels.

The increase in O content observed with dopant level may reflect oxygen-rich RE-oxide inclusions (e.g., Sm_2_O_3_) or the formation of hydroxides, such as La(OH)_3_ in La:ZnO, as also evidenced by XRD. Similar compositional validation was obtained for La:ZnO and Er:ZnO films, with atomic percentages matching the nominal values and confirming the successful incorporation of RE dopants in all cases. The EDX analysis supports the key findings from the morphological and structural investigations, namely that RE dopants are effectively introduced into the ZnO matrix up to moderate concentrations, with uniform distribution. At higher doping levels, partial phase segregation occurs, especially for Er and Sm, leading to the formation of RE-rich nanodomains that may influence charge transport and optical absorption.

### 3.5. Optical Properties: UV-Vis Absorption and Bandgap Analysis

**Figure 5:**
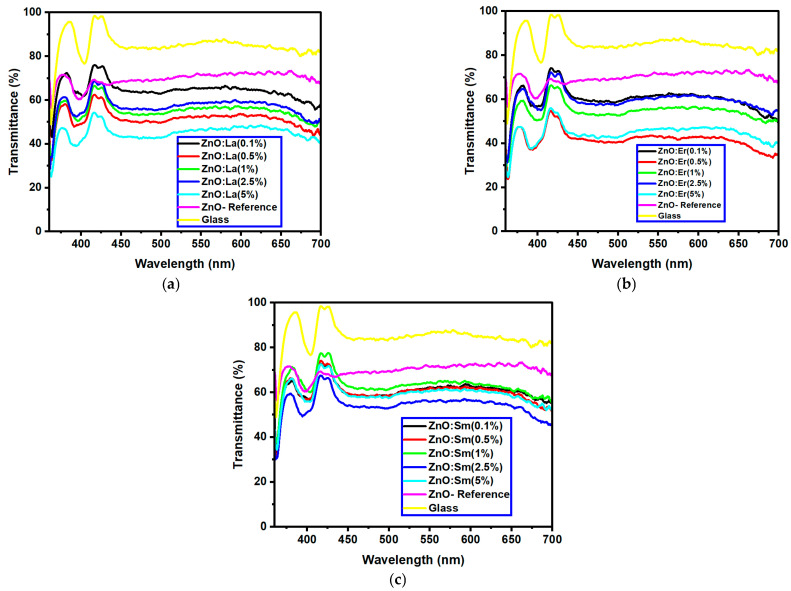
Optical transmittance UV-Vis spectra of different dopant concentrations for RE:ZnO thin films: (**a**) La:ZnO, (**b**) Er:ZnO, and (**c**) Sm:ZnO.

The optical transmittance and absorption behavior of the RE:ZnO thin films were analyzed by UV-Vis spectroscopy in the wavelength range of 300–900 nm. All samples exhibited high optical transparency, with average transmittance values around 70% in the visible region as it can be observed in Figure 5, confirming their potential applicability in transparent optoelectronic devices.

The influence of rare-earth (RE) dopants—La, Er, and Sm—on the optical bandgap of ZnO thin films was systematically evaluated by comparing Eg values at matched concentrations (0.1%, 0.5%, 1%, 2.5%, and 5%). All three RE dopants induced bandgap narrowing compared to undoped ZnO (Eg = 3.341 eV), but their efficiency and behavior varied depending on the dopant type, as can be observed from Table 4.

To evaluate the effect of doping on the electronic structure, the optical bandgap (Eg) was extracted from the absorption spectra using the Tauc method for direct allowed transitions:

(αhν)^2^ = A(hν − Eg) (1)

where α is the absorption coefficient, hν the photon energy, and A a constant. Eg was determined by extrapolating the linear portion of the Tauc plots to the energy axis (i.e., where (αhν)^2^ = 0). ***All the Tauc plots are presented in*** Appendix B.

**La:ZnO** films showed gradual bandgap narrowing with increasing dopant content. Eg decreased from 2.964 eV (0.1% La) to 2.930 eV (5% La), which can be attributed to the formation of localized states near the conduction band minimum and the increase in free carrier density induced by La^3+^ incorporation. The relatively consistent Eg values suggest that the La dopant acts primarily by introducing shallow donor states, without disrupting the overall band structure at low-to-moderate concentrations. **Er:ZnO** exhibited a more complex trend, with Eg values ranging from 2.940 to 2.970 eV. The maximum Eg of 2.970 eV was observed at 2.5% Er, likely due to quantum confinement or Burstein–Moss effects, while slight bandgap narrowing occurred at higher doping (5%), possibly due to the formation of Er_2_O_3_ and associated defect states. These fluctuations reflect a balance between two competing mechanisms: band filling (leading to apparent Eg widening) and defect-state formation (causing bandgap narrowing). **Sm:ZnO** samples showed the most stable optical response, with Eg values tightly clustered between 2.965 and 2.971 eV across all concentrations. This suggests that Sm^3+^ ions exert a moderate influence on the electronic structure of ZnO and that their incorporation does not substantially perturb the band edges. However, at the highest Sm content (5%), the slightly lower Eg (2.965 eV) may result from structural disorder and the onset of Sm_2_O_3_ phase formation, in agreement with the XRD and Raman findings.

These results confirm that RE doping enables fine-tuning of the ZnO optical bandgap without severely compromising transparency. While bandgap modulation is modest in magnitude, it is directionally consistent with the changes in crystallinity, microstrain, and defect levels discussed in Section 3.2 and Section 3.3.

Overall, the observed bandgap values (2.93–2.97 eV) are lower than those typically reported for RE:ZnO films deposited by other techniques (e.g., spray pyrolysis and sol–gel), which may be attributed to the unique nanostructured morphology and defect chemistry imparted by the electrospun–calcined microcluster precursors. These characteristics could be advantageous for light absorption enhancement and photoconductive behavior in UV or visible-light optoelectronic devices.

### 3.6. Comparative Discussion and Application Relevance

The comprehensive analysis of RE:ZnO thin films synthesized using nanostructured microclusters derived from electrospinning–calcination highlights both the versatility and the tunability offered by La^3+^, Er^3+^, and Sm^3+^ doping. Table 5 summarizes the main trends in structural, morphological, and optical properties, offering a direct comparison among the three dopants and pure ZnO.

These results demonstrate that the type and concentration of RE dopants directly influence the final thin film characteristics:

**La^3+^** promotes nanorod growth and lattice distortion, with moderate bandgap narrowing, and the late formation of La(OH)_3_. La:ZnO films offer tunable optical gaps with well-formed crystallites, making them promising for UV detectors and transparent thin-film transistors.

**Er^3+^** favors particle growth and phase segregation at relatively low concentrations, resulting in larger grain sizes and a complex phononic signature. Er:ZnO films may be useful in photonic or luminescent applications where Er^3+^ f-levels can be exploited.

**Sm^3+^** yields elongated nanorods and subtle Eg modulation, with well-preserved ZnO phonon modes at low doping. The strong anisotropy and Sm_2_O_3_ phase formation at high concentrations make Sm:ZnO attractive for polarization-sensitive devices or anisotropic sensors.

Compared to conventional ZnO thin films fabricated by sol–gel, spray pyrolysis, or CVD methods, the electrospun-derived RE:ZnO films present unique advantages: (i) **tailored nanoscale morphology** from pre-structured microclusters; (ii) **moderate doping without harsh chemical precursors**; and (iii) **compatibility with low-temperature, drop-cast deposition**, enabling integration on flexible substrates.

The key limitation remains the moderate adhesion to glass substrates, which currently restricts device integration. However, this drawback can be addressed in future work by introducing optically inert adhesion layers or sol–gel binders that preserve transparency while improving film robustness.

## 4. Conclusions

This work presents a novel approach to fabricating nanostructured ZnO and rare-earth-doped ZnO (La^3+^, Er^3+^, and Sm^3+^) thin films by integrating two scalable and cost-effective techniques: electrospinning–calcination and drop-casting. Unlike conventional vapor-phase or wet-chemical methods, this strategy leverages pre-engineered doped ZnO microclusters with tailored morphologies and controlled dopant content, enabling a unique transfer of nanoscale features into thin-film architecture without the need for in situ high-temperature synthesis or complex precursor chemistry.

A comprehensive comparative analysis across dopant types and concentrations (0.1–5 at.%) reveals distinct and dopant-specific effects on microstructure, crystallinity, vibrational modes, and optical properties. La doping favors nanorod growth and induces mild bandgap narrowing; Er enhances grain size and triggers secondary phase formation at lower concentrations; and Sm promotes anisotropic rod-like growth and introduces complex Raman activity. Despite moderate adhesion on glass substrates, all films demonstrate uniform RE distribution, high transparency (~70%), and bandgap values in the 2.93–2.97 eV range, reinforcing their applicability for transparent optoelectronic devices, UV detectors, and future flexible sensing platforms. The scientific originality of this study lies in the integration of electrospun nanostructured materials into thin films—a route largely unexplored in the context of doped ZnO—and in the systematic comparison of three chemically and structurally distinct rare-earth ions. Moreover, the synthesis methodology is adaptable, scalable, and compatible with flexible substrates, aligning well with current priorities in low-cost, solution-processed, and sustainable nanomaterial development. The **added value** of this approach lies in the use of electrospun nanostructured microclusters as precursors, which results in films combining fine-tuned morphology, controlled defect structure, and adjustable optical properties. Compared to films produced by conventional spray pyrolysis or sol–gel methods, these materials exhibit enhanced light–matter interaction potential, advantageous for devices requiring visible-light responsiveness such as photodetectors, solar cells, and electro-optical modulators.

In the current scientific landscape—where the demand for defect-engineered, nanostructured metal oxides with tailored functionalities is rapidly growing—this work provides timely and relevant insight into both the fabrication and fundamental understanding of RE:ZnO systems. It sets a strong foundation for further device-level integration and opens promising pathways toward multifunctional thin films engineered from electrospun nanomaterials. Nonetheless, the present films show **limited adhesion** to glass substrates, a factor that must be addressed before integration into robust device architectures. While their optoelectronic properties fulfill key functional requirements, mechanical durability can be improved in future work by introducing optically inactive binders or adhesion-promoting interlayers. Another limitation is the lack of in-device testing, which will be essential to translate the observed laboratory-scale properties into real-world performance metrics.

**Future perspectives** include the following:

(i) Optimizing deposition parameters and substrate treatments to improve adhesion and film continuity;
(ii) Extending the doping strategy to other rare-earth and transition-metal ions to further tune optical and electronic behavior;
(iii) Integrating the films into prototype optoelectronic devices to assess their functional advantages over conventional ZnO;
(iv) Performing in situ studies under operational conditions to better understand stability, defect dynamics, and performance evolution.

By addressing these aspects, the current findings establish a foundation for the rational design of nanostructured ZnO-based thin films with tailored properties for advanced optoelectronic, sensing, and photocatalytic applications.

## Figures and Tables

**Table 1:** Crystallite size and microstrain determined by Rietveld refinement for RE:ZnO thin films.

Sample	τ_ZnO (nm)	τ_Secondary (nm)	ε_ZnO (%)	ε_Secondary (%)
ZnO (undoped)	23.3	—	+0.06	—
0.1% La:ZnO	23.9	—	+0.18	—
1% La:ZnO	24.8	—	+0.24	—
2.5% La:ZnO	24.4	12.5	+0.20	+0.32
5% La:ZnO	22.9	14.9	+0.16	+0.29
0.1% Er:ZnO	21.0	—	+0.09	—
0.5% Er:ZnO	23.2	—	+0.21	—
1% Er:ZnO	20.8	—	+0.16	—
2.5% Er:ZnO	23.2	8.9	+0.25	+0.36
5% Er:ZnO	21.2	9.2	+0.21	+0.28
0.1% Sm:ZnO	22.0	—	+0.16	—
0.5% Sm:ZnO	22.7	—	+0.23	—
1% Sm:ZnO	21.2	—	+0.20	—
2.5% Sm:ZnO	21.9	9.2	+0.15	+0.26
5% Sm:ZnO	22.5	12.3	+0.19	+0.24

**Table 2:** Extracted Raman spectral features of pure and RE-doped ZnO thin films (E_2_ (high) phonon position, FWHM trend, and dopant-induced modes).

Sample	E_2_ (high) Position (cm^−1^)	FWHM (Qualitative)	Defect/Secondary Modes
Pure ZnO	437.0	Sharp	None
La:ZnO 0.1%	436.8	Slightly broadened	Weak ~580
La:ZnO 0.5%	436.2	Moderate	580
La:ZnO 1%	435.6	Moderate	580, ~330
La:ZnO 2.5%	435.0	Broad	580, ~330
La:ZnO 5%	434.8	Broad	580, ~330
Er:ZnO 0.1%	436.7	Slightly broadened	580
Er:ZnO 0.5%	435.9	Moderate	580, ~655
Er:ZnO 1%	435.0	Broad	580, 655, 330
Er:ZnO 2.5%	434.2	Very broad	580, 655, 330
Er:ZnO 5%	434.0	Very broad	580, 655, 330
Sm:ZnO 0.1%	436.6	Slightly broadened	Weak ~580
Sm:ZnO 0.5%	435.6	Moderate	580, ~610
Sm:ZnO 1%	434.9	Moderate	580, 610, 330
Sm:ZnO 2.5%	434.2	Broad	580, 610, 330
Sm:ZnO 5%	434.0	Very broad	580, 610, 330

**Table 3:** Elemental composition of **La:ZnO, Er:ZnO, and Sm:ZnO** thin films determined by EDX analysis (all the values have ±5% errors).

Sample	Element	Wt.%	At.%	Sample	Element	Wt.%	At.%	Sample	Element	Wt.%	At.%
**0.1% La:ZnO**	O_k_	15	14.8	**0.1% Er:ZnO**	O_k_	19.22	49.35	**0.1% Sm: ZnO**	O_k_	10.51	32.96
	Zn_k_	84.84	58.13		Er_L_	0.32	0.08		Sm_L_	2.41	0.60
	La_L_	0.23	0.08		Zn_k_	80.47	50.58		Zn_k_	87.08	66.44
**0.5% La:ZnO**	O_k_	14	41.80	**0.5% Er:ZnO**	O_k_	16.36	44.64	**0.5% Sm: ZnO**	O_k_	11.72	35.56
	Zn_k_	83.88	58.49		Er_L_	1.20	0.31		Sm_L_	2.60	0.84
	La_L_	1.75	0.57		Zn_k_	82.44	55.05		Zn_k_	85.68	35.56
**1% La:ZnO**	O_k_	17	45.35	**1% Er:ZnO**	O_k_	14.35	41.25	**1% Sm: ZnO**	O_k_	11.86	35.98
	Zn_k_	80.48	53.74		Er_L_	3.52	0.97		Sm_L_	3.42	1.11
	La_L_	2.90	0.91		Zn_k_	82.13	57.78		Zn_k_	84.72	62.92
**2.5% La:ZnO**	O_k_	15	42.34	**2.5% Er:ZnO**	O_k_	13.87	41.17	**2.5% Sm: ZnO**	O_k_	13.03	39.17
	Zn_k_	77.66	55.08		Er_L_	8.41	2.38		Sm_L_	7.56	2.42
	La_L_	7.73	2.58		Zn_k_	77.72	56.45		Zn_k_	79.41	58.41
**5% La:ZnO**	O_k_	18	50.22	**5% Er:ZnO**	O_k_	17.72	50.17	**5% Sm: ZnO**	O_k_	13.65	41.52
	Zn_k_	60.57	42.54		Er_L_	17.00	4.60		Sm_L_	13.78	4.46
	La_L_	21.92	7.24		Zn_k_	65.28	45.23		Zn_k_	72.57	54.02

**Table 4:** Optical bandgap (Eg) values for RE:ZnO thin films.

Dopant	Concentration (at.%)	Eg (eV)
None	0.0	3.34
La	0.1	2.96
La	0.5	2.95
La	1.0	2.95
La	2.5	2.96
La	5.0	2.93
Er	0.1	2.96
Er	0.5	2.94
Er	1.0	2.96
Er	2.5	2.97
Er	5.0	2.94
Sm	0.1	2.97
Sm	0.5	2.97
Sm	1.0	2.97
Sm	2.5	2.97
Sm	5.0	2.97

**Table 5:** Summary of main observations for the RE:ZnO-doped thin films.

Property/Sample	Pure ZnO	La:ZnO	Er:ZnO	Sm:ZnO
**Morphology**	Nanoparticles, uniform	Nanoparticles + nanorods at all concentrations; longest at 5%	Only spherical particles; increased coalescence with concentration	Nanoparticles + pronounced nanorods at ≥2.5%
**Particle Size (nm)**	~50–60	54.4 → 69.4 (max at 1%: 86.6)	66.9 → 133.1 (monotonic increase)	42.3 → 84.1 (sharp increase at 5%)
**Nanorod Length (nm)**	None	327.5 → 442.3	Not observed	291.8 → 938.6
**Secondary Phases**	None	La(OH)_3_ at ≥2.5%	Er_2_O_3_ at ≥1%	Sm_2_O_3_ at ≥2.5%
**Crystallite Size (nm)**	23.3	~24.8 (ZnO), ~15 (La(OH)_3_)	~21 (ZnO), ~9 (Er_2_O_3_)	~22 (ZnO), ~12 (Sm_2_O_3_)
**Microstrain (%)**	+0.06	+0.24 (ZnO)/+0.29 (secondary)	+0.21/+0.28	+0.19/+0.24
**Raman E_2_ (high) peak**	Strong, sharp	Broadened and weakened at ≥1%	Shifted, suppressed at ≥2.5%	Slightly broadened; defect-related modes appear at ≥2.5%
**New Raman modes**	None	La-induced at ~391 and ~1150 cm^−1^	Er–O and Er_2_O_3_ bands at 150–800 cm^−1^	Sm_2_O_3_ modes at 490, 678 cm^−1^
**Eg (eV)**	~2.97	2.96 → 2.93 (decrease with conc.)	2.96 → 2.97 (non-monotonic)	2.97 → 2.97 (stable)
**Transparency (Vis)**	~70%	~70%	~70%	~70%
**EDX Distribution of dopant**	—	Uniform	Uniform	Uniform
**Film Adhesion**	Moderate	Moderate	Moderate	Moderate

## Data Availability

The datasets used and/or analyzed during the current study are available from the corresponding author M.P.S. (mirasuchea@gmail.com; mira.suchea@imt.ro; or mirasuchea@hmu.gr) due to the fact that the funding project is ongoing and they are part of a larger restricted yet database.

## Abbreviations

The following abbreviations are used in this manuscript:

ZnO	Zinc Oxide
RE	Rare-Earth
La	Lanthanum
Er	Erbium
Sm	Samarium
XRD	X-ray Diffraction
SEM	Scanning Electron Microscopy
EDX	Energy Dispersive X-ray Spectroscopy
UV–Vis	Ultraviolet–Visible Spectroscopy
PL	Photoluminescence
Eg	Optical Bandgap Energy
λ	Wavelength
D	Crystallite Size
ε	Microstrain
δ	Dislocation Density
τ_ZnO_	Crystallite Size of ZnO phase from Rietveld refinement
τ_secondary_	Crystallite Size of Secondary Phase
ε_ZnO_	Microstrain of ZnO phase from Rietveld refinement
ε_secondary_	Microstrain of Secondary Phase
nm	Nanometer
eV	Electron Volt
at.%	Atomic Percent
Tauc plot	Graphical Method to Estimate Bandgap from UV–Vis Absorption
O_2_	Molecular Oxygen
RE:ZnO	Rare-Earth-Doped ZnO Thin Film

## Appendix A

Dimensional distribution of surface feature evaluation.

**Table A1:** Dimensional distribution and grain size evaluation of La:ZnO thin films’ surface grains.

Sample Films	Nanoparticles	Nanorods (nm)	
		Length	Width
0.1% La:ZnO			
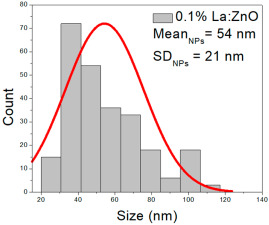
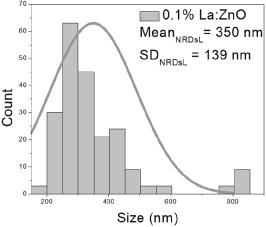
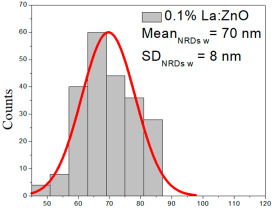
0.5% La:ZnO			
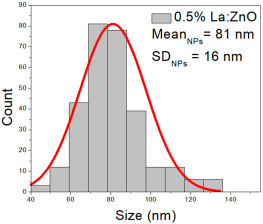
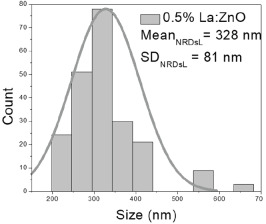
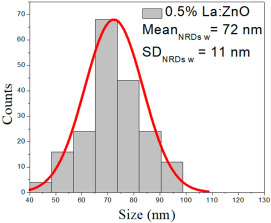
1% La:ZnO			
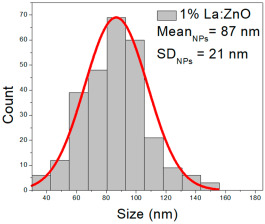
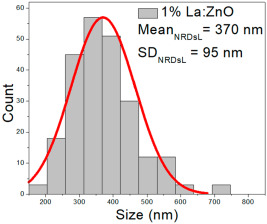
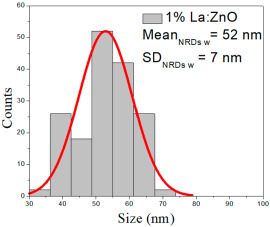
2.5% La:ZnO			
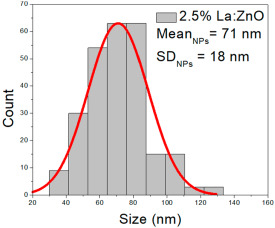
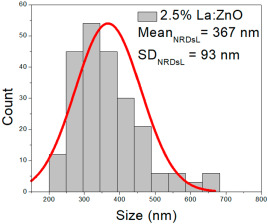
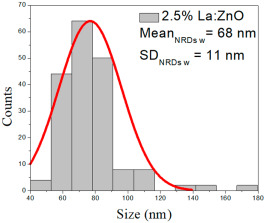
5% La:ZnO			
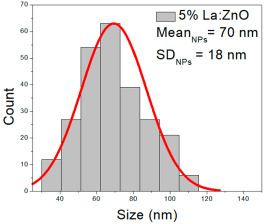
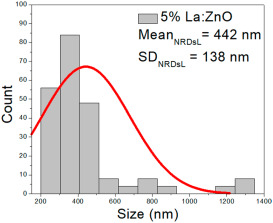
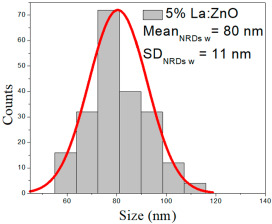

**Table A2:** Particle size evaluation for La:ZnO thin films’ surface grains.

Sample Film	NPS			NRDs—Length			NRDs—Width		
	Min–Max (nm)	FWHM (nm)	Mean ± SD (nm)	Min–Max (nm)	FWHM (nm)	Mean ± SD (nm)	Min–Max (nm)	FWHM (nm)	Mean ± SD (nm)
**0.1%** **La:ZnO**	25–109	34–75	**54 ± 21**	194–850	227–490	**350 ± 139**	50–86	60–79	**70 ± 8**
**0.5%** **La:ZnO**	45–134	62–98	**81 ± 16**	202–638	242–415	**328 ± 81**	45–98	60–85	**72 ± 11**
**1%** **La: ZnO**	38–147	64–110	**87 ± 21**	200–700	273–472	**370 ± 95**	35–70	43–61	**52 ± 7**
**2.5%** **La:ZnO**	37–127	50–90	**71 ± 18**	205–660	262–470	**367 ± 93**	41–117	58–96	**68 ± 11**
**5%** **La: ZnO**	36–110	50–88	**70 ± 18**	225–1309	230–652	**442 ± 138**	68–93	57–110	**80 ± 11**

The SEM analysis of La-doped ZnO films with varying La concentrations (0.1%, 0.5%, 1%, 2.5%, and 5%) revealed two primary morphologies: predominantly spherical nanoparticles (NPs) and nanorod (NRD) structures. The distribution of spherical NPs and NRDs was quantitatively assessed through SEM images by measuring approximately 200 particles and nanorod lengths per sample. ImageJ 1.54 m (open-source software) was used for data extraction and analysis, allowing for the construction of corresponding histograms. Gaussian fitting of the histograms provided robust characterization of particle and nanorod size distributions.

The detailed size distribution data are summarized below:

**0.1% La:ZnO** films showed NP sizes ranging from 25 to 109 nm (mean 54 ± 21 nm, FWHM: 34–75 nm), and NRD lengths of 194–850 nm (mean 350 ± 139 nm, FWHM: 227–490 nm) and widths of 50–86 nm (mean 70 ± 8 nm, FWHM: 60–79 nm).

**0.5% La:ZnO** films had NPs from 45 to 134 nm (81 ± 16 nm, FWHM: 62–98 nm), and NRDs with lengths of 202–638 nm (328 ± 81 nm, FWHM: 242–415 nm) and widths of 45–98 nm (72 ± 11 nm, FWHM: 60–85 nm).

**1% La:ZnO** films exhibited NPs between 38 and 147 nm (87 ± 21 nm, FWHM: 64–110 nm), and NRD lengths of 200–700 nm (370 ± 95 nm, FWHM: 273–472 nm) and widths of 35–70 nm (52 ± 7 nm, FWHM: 43–61 nm).

**2.5% La:ZnO** films showed NPs ranging from 37 to 127 nm (71 ± 18 nm, FWHM: 50–90 nm), and NRD lengths of 205–660 nm (367 ± 93 nm, FWHM: 262–470 nm) and widths of 41–117 nm (68 ± 11 nm, FWHM: 58–96 nm).

**5% La:ZnO** films presented NPs of 36–110 nm (70 ± 18 nm, FWHM: 50–88 nm), and NRD lengths from 225 to 1309 nm (442 ± 138 nm, FWHM: 230–652 nm) and widths of 68–93 nm (80 ± 11 nm, FWHM: 57–110 nm).

These observations indicate morphological evolution with La doping, as reflected in increasing average NP size, broadening of nanorod length distributions, and moderate fluctuations in nanorod widths, particularly at higher La concentrations.

**Table A3:** Dimensional distribution and grain size evaluation of Er:ZnO thin films’ surface grains.

Sample Films	Nanoparticles	Nanorods (nm)	
		Length	Width
0.1% Er:ZnO			
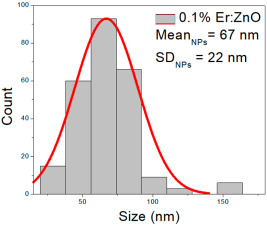
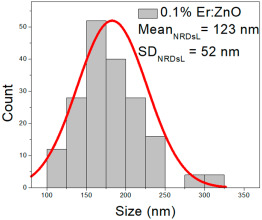
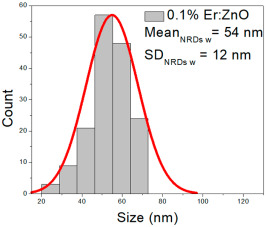
0.5% Er:ZnO			
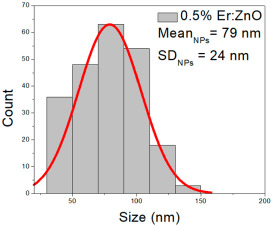
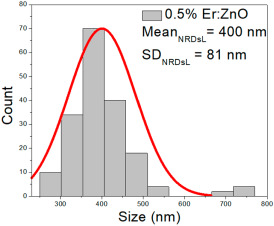
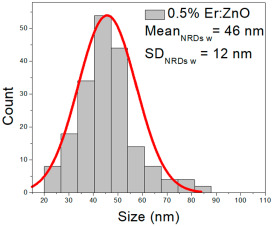
1% Er:ZnO			
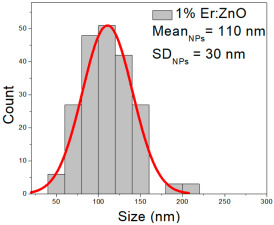
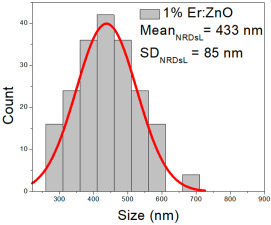
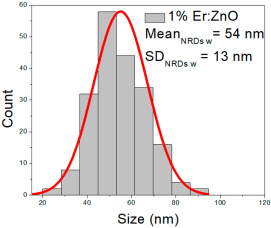
2.5% Er:ZnO			
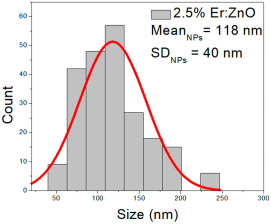
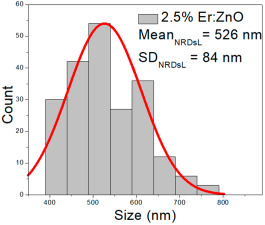
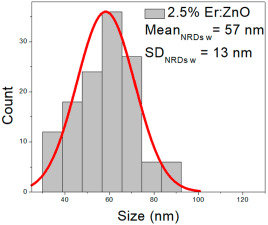
5% Er:ZnO			
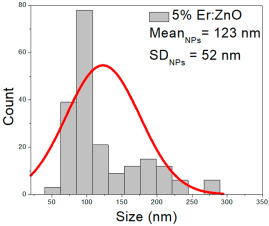
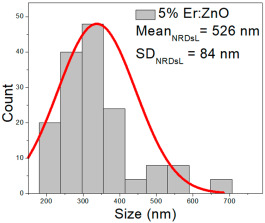
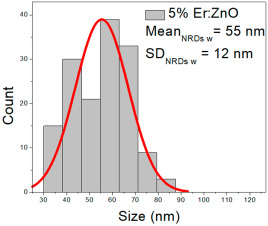

**Table A4:** Particle size evaluation for Er:ZnO thin films’ surface grains.

Sample Film	NPS			NRDs—Length			NRDs—Width		
	Min–Max (nm)	FWHM (nm)	Mean ± SD (nm)	Min–Max (nm)	FWHM (nm)	Mean ± SD (nm)	Min–Max (nm)	FWHM (nm)	Mean ± SD (nm)
**0.1% Er:ZnO**	24–160	43–90	**70 ± 22**	110–324	132–232	**182 ± 44**	27–102	40–70	**54 ± 12**
**0.5% Er:ZnO**	38–135	52–107	**79 ± 24**	270–763	308–493	**400 ± 81**	22–82	32–58	**46 ± 12**
**1% Er: ZnO**	43–204	75–145	**110 ± 30**	269–701	340–530	**433 ± 85**	23–93	41–68	**54 ± 13**
**2.5% Er:ZnO**	50–230	75–160	**118 ± 40**	373–778	434–618	**526 ± 84**	31–90	42–73	**57 ± 13**
**5% Er: ZnO**	62–283	66–173	**123 ± 52**	176–652	227–448	**337 ± 106**	35–81	42–67	**55 ± 12**

The SEM analysis of Er-doped ZnO films with varying Er concentrations (0.1%, 0.5%, 1%, 2.5%, and 5%) revealed two primary morphologies: predominantly spherical nanoparticles (NPs) and nanorod (NRD) structures. The distribution of spherical NPs and NRDs was quantitatively assessed through SEM images by measuring approximately 200 particles and nanorod lengths per sample. ImageJ (open-source software) was used for data extraction and analysis, allowing for the construction of corresponding histograms. Gaussian fitting of the histograms provided robust characterization of particle and nanorod size distributions.

The results are summarized as follows:

**0.1% Er:ZnO** films showed NP diameters ranging from 24 to 160 nm (mean 70 ± 22 nm, FWHM: 43–90 nm), and NRD lengths of 110–324 nm (mean 182 ± 44 nm, FWHM: 132–232 nm) and widths of 27–102 nm (mean 54 ± 12 nm, FWHM: 40–70 nm).

**0.5% Er:ZnO** films exhibited NPs of 38–135 nm (79 ± 24 nm, FWHM: 52–107 nm), and NRDs with lengths of 270–763 nm (400 ± 81 nm, FWHM: 308–493 nm) and widths of 22–82 nm (46 ± 12 nm, FWHM: 32–58 nm).

**1% Er:ZnO** films contained NPs from 43 to 204 nm (110 ± 30 nm, FWHM: 75–145 nm), and NRD lengths of 269–701 nm (433 ± 85 nm, FWHM: 340–530 nm) and widths of 23–93 nm (54 ± 13 nm, FWHM: 41–68 nm).

**2.5% Er:ZnO** films showed NPs of 50–230 nm (118 ± 40 nm, FWHM: 75–160 nm), and NRDs with lengths of 373–778 nm (526 ± 84 nm, FWHM: 434–618 nm) and widths of 31–90 nm (57 ± 13 nm, FWHM: 42–73 nm).

**5% Er:ZnO** films presented NPs between 62–283 nm (123 ± 52 nm, FWHM: 66–173 nm), and NRD lengths of 176–652 nm (337 ± 106 nm, FWHM: 227–448 nm) and widths of 35–81 nm (55 ± 12 nm, FWHM: 42–67 nm).

This analysis highlights the morphological evolution of Er:ZnO films with increasing Er content, indicating a growth trend in both nanoparticle and nanorod dimensions, along with broader size distributions at higher doping levels.

**Table A5:** Dimensional distribution and grain size evaluation of Sm:ZnO thin films’ surface grains.

Sample Films	Nanoparticles	Nanorods (nm)	
		Length	Width
**0.1% Sm:ZnO**			
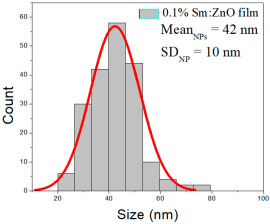
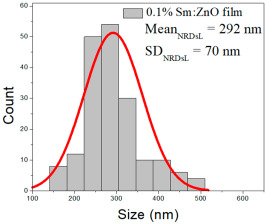
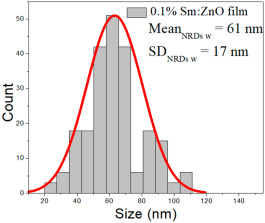
**0.5% Sm:ZnO**			
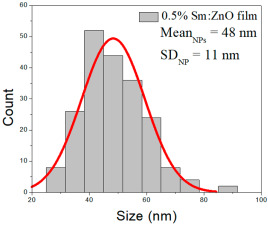
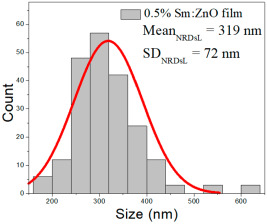
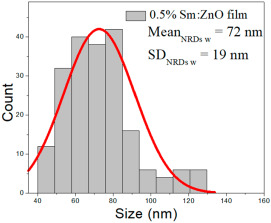
**1% Sm:ZnO**			
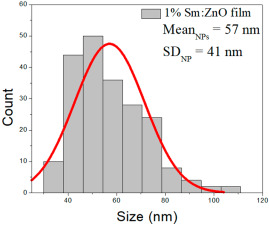
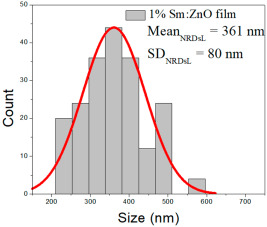
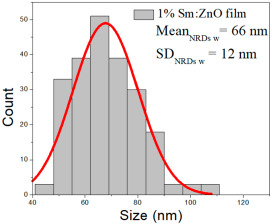
**2.5% Sm:ZnO**			
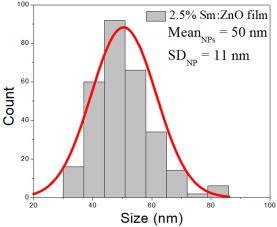
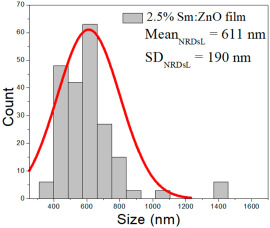
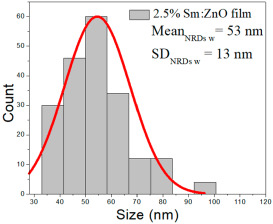
**5% Sm:ZnO**			
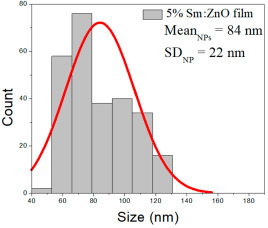
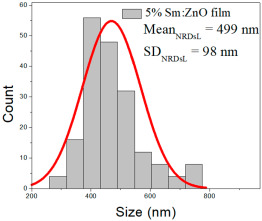
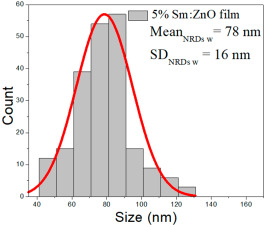

**Table A6:** Particle size evaluation for Sm:ZnO thin films’ surface grains.

Sample Film	NPS			NRDs—Length			NRDs—Width		
	Min–Max (nm)	FWHM (nm)	Mean ± SD (nm)	Min–Max (nm)	FWHM (nm)	Mean ± SD (nm)	Min–Max (nm)	FWHM (nm)	Mean ± SD (nm)
**0.1% Sm:ZnO**	23–75	32–53	**42 ± 10**	281–494	220–358	**292 ± 70**	22–108	46–80	**61 ± 17**
**0.5% Sm:ZnO**	27–87	37–60	**48 ± 11**	193–635	240–390	**319 ± 72**	41–73	55–93	**72 ± 19**
**1% Sm: ZnO**	32–105	42–73	**57 ± 14**	216–578	282–442	**361 ± 80**	44–110	54–81	**66 ± 12**
**2.5% Sm:ZnO**	24–111	38–63	**50 ± 11**	360–1442	418–804	**611 ± 190**	33–100	40–66	**53 ± 13**
**5% Sm: ZnO**	51–188	60–108	**84 ± 22**	301–744	365–564	**499 ± 98**	42–128	60–95	**78 ± 16**

The SEM analysis of Sm-doped ZnO films with varying Sm concentrations (0.1%, 0.5%, 1%, 2.5%, and 5%) revealed two primary morphologies: predominantly spherical nanoparticles (NPs) and nanorod (NRD) structures. The distribution of spherical NPs and NRDs was quantitatively assessed through SEM images by measuring approximately 200 particles and nanorod lengths per sample. ImageJ (open-source software) was used for data extraction and analysis, allowing for the construction of corresponding histograms. Gaussian fitting of the histograms provided robust characterization of particle and nanorod size distributions.

The detailed measurements are as follows:

**0.1% Sm:ZnO** films exhibited NP sizes ranging from 23 to 75 nm (mean 42 ± 10 nm, FWHM: 32–53 nm), and NRD lengths from 281–494 nm (mean 292 ± 70 nm, FWHM: 220–358 nm) and widths of 22–108 nm (mean 61 ± 17 nm, FWHM: 46–80 nm).

**0.5% Sm:ZnO** films had NPs of 27–87 nm (48 ± 11 nm, FWHM: 37–60 nm), and NRD lengths of 193–635 nm (319 ± 72 nm, FWHM: 240–390 nm) and widths of 41–73 nm (72 ± 19 nm, FWHM: 55–93 nm).

**1% Sm:ZnO** films showed NP sizes from 32 to 105 nm (57 ± 14 nm, FWHM: 42–73 nm), and NRD lengths of 216–578 nm (361 ± 80 nm, FWHM: 282–442 nm) and widths of 44–110 nm (66 ± 12 nm, FWHM: 54–81 nm).

**2.5% Sm:ZnO** films displayed NPs in the range of 24–111 nm (50 ± 11 nm, FWHM: 38–63 nm), and NRDs with lengths from 360 to 1442 nm (611 ± 190 nm, FWHM: 418–804 nm) and widths of 33–100 nm (53 ± 13 nm, FWHM: 40–66 nm).

**5% Sm:ZnO** films contained NPs between 51 and 188 nm (84 ± 22 nm, FWHM: 60–108 nm), and NRDs with lengths from 301–744 nm (499 ± 98 nm, FWHM: 365–564 nm) and widths ranging from 42 to 128 nm (78 ± 16 nm, FWHM: 60–95 nm).

These results suggest that increasing Sm concentration influences both particle and rod morphologies, particularly resulting in broader nanorod length distributions and a noticeable increase in average NP size at higher doping levels.

### Elemental Composition and Dopant Distribution (EDX Analysis)

**Table A7:** Elemental composition and dopant distribution of La:ZnO thin films.

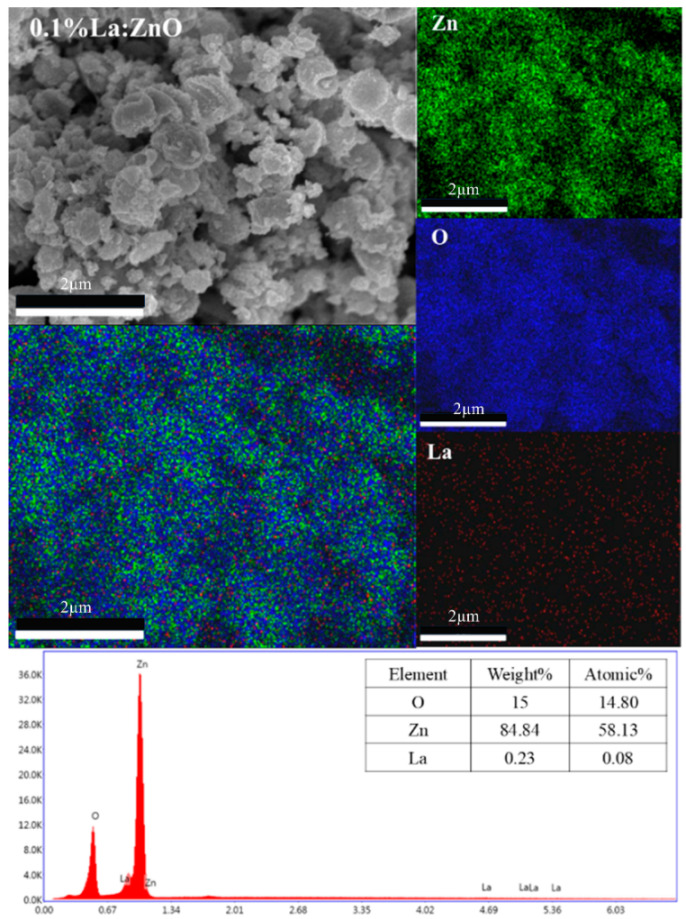
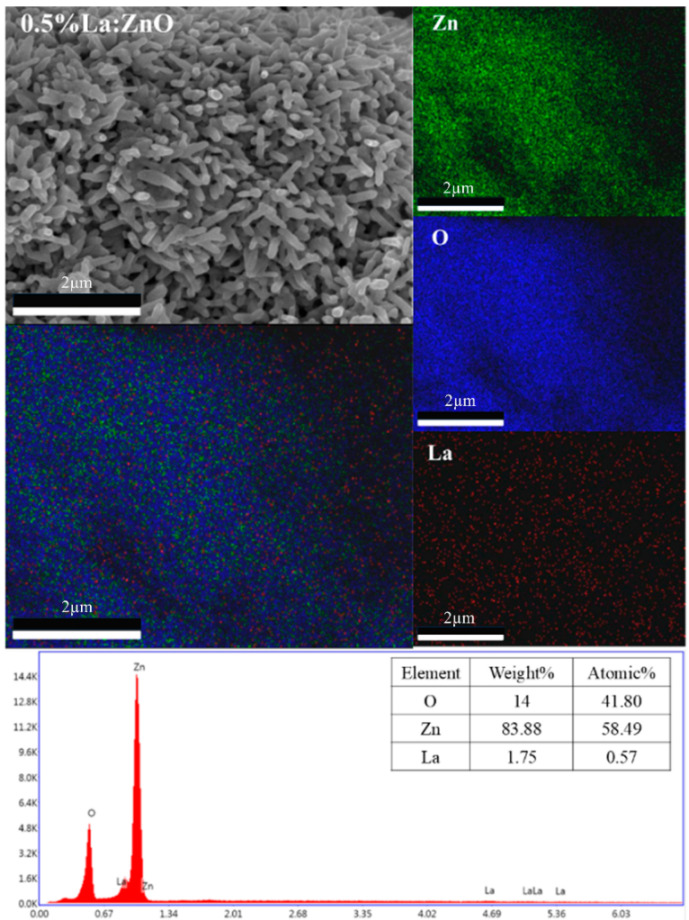

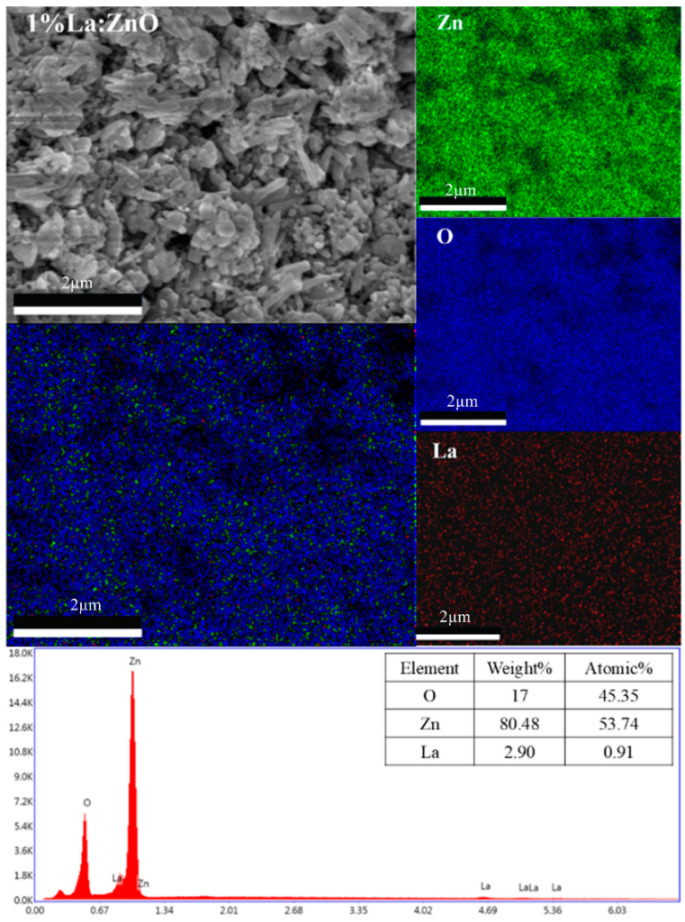
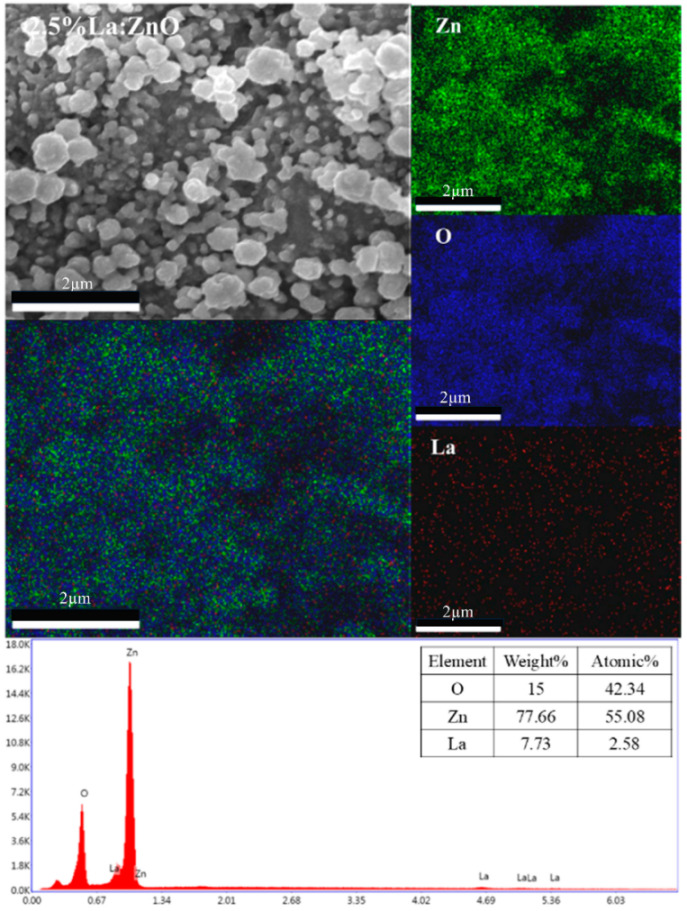

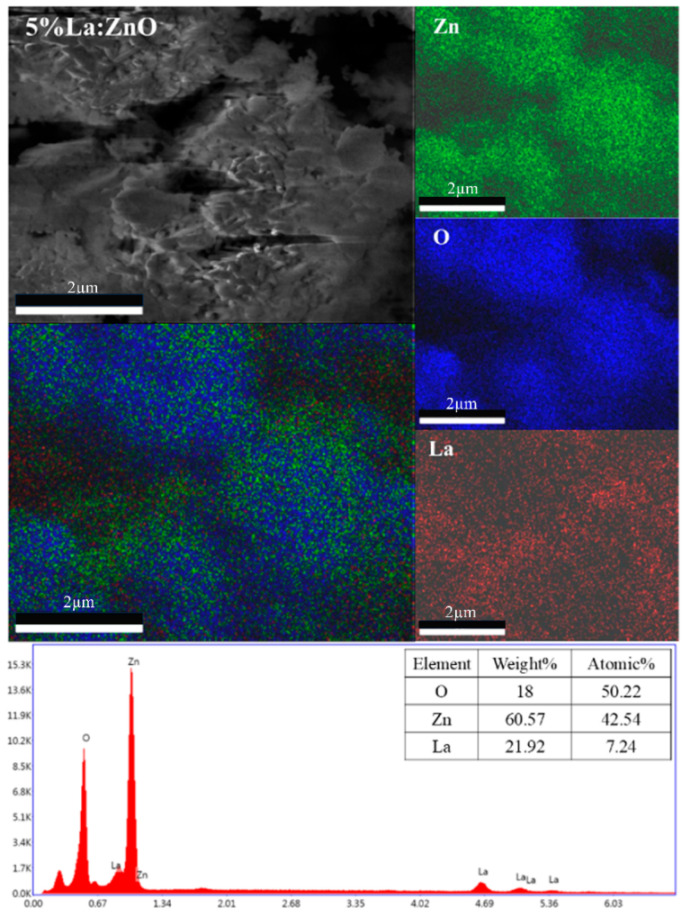

	
	
	
**Sample**	**Element**	**Weight%**	**Atomic%**
**0.1% La-doped ZnO**	O_k_	15	14.8
	Zn_k_	84.84	58.13
	**La_L_**	0.23	**0.08**
**0.5% La-doped ZnO**	O_k_	14	41.80
	Zn_k_	83.88	58.49
	**La_L_**	1.75	**0.57**
**1% La-doped ZnO**	O_k_	17	45.35
	Zn_k_	80.48	53.74
	**La_L_**	2.90	**0.91**
**2.5% La-doped ZnO**	O_k_	15	42.34
	Zn_k_	77.66	55.08
	**La_L_**	7.73	**2.58**
**5% La-doped ZnO**	O_k_	18	50.22
	Zn_k_	60.57	42.54
	**La_L_**	21.92	**7.24**

**Table A8:** Elemental composition and dopant distribution of Er:ZnO thin films.

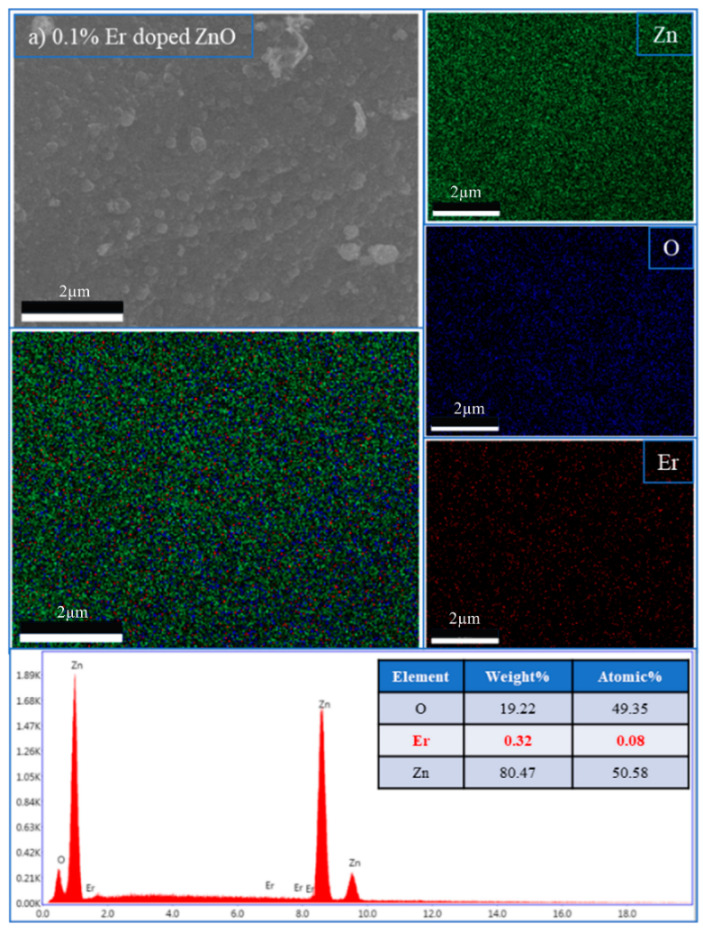
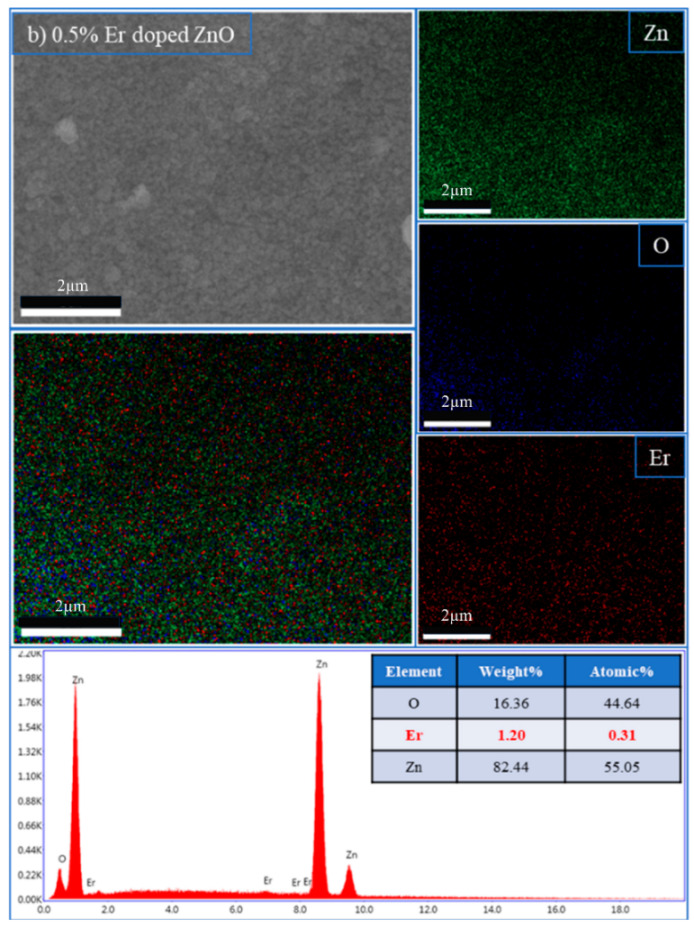

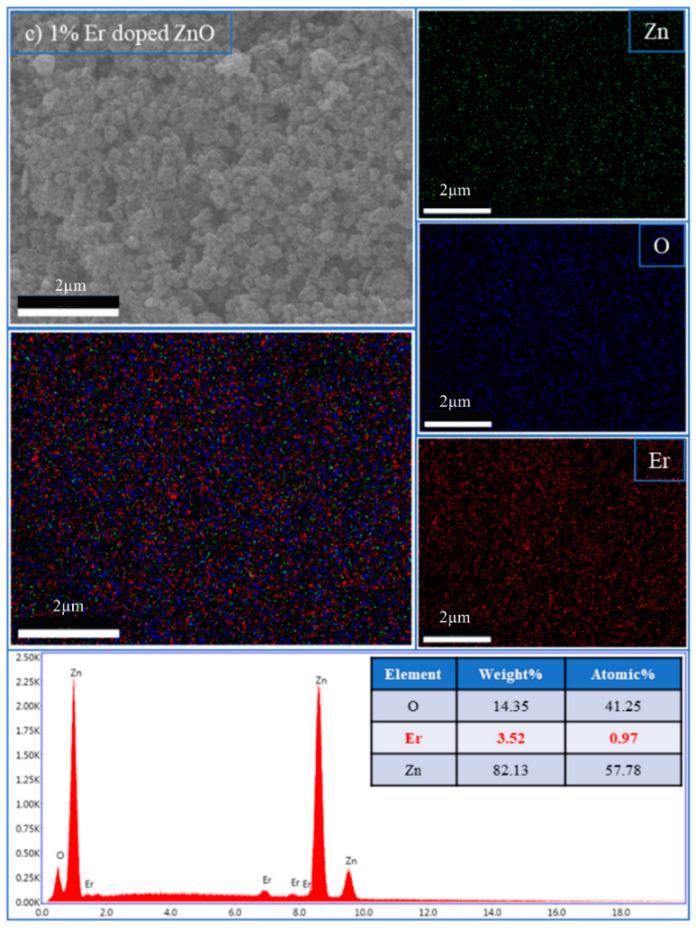
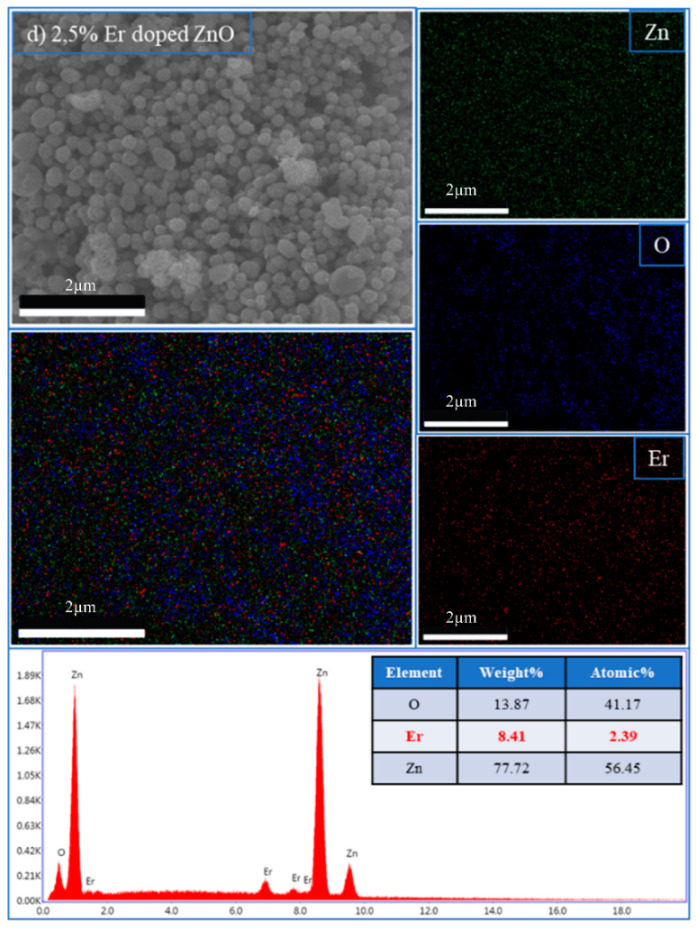

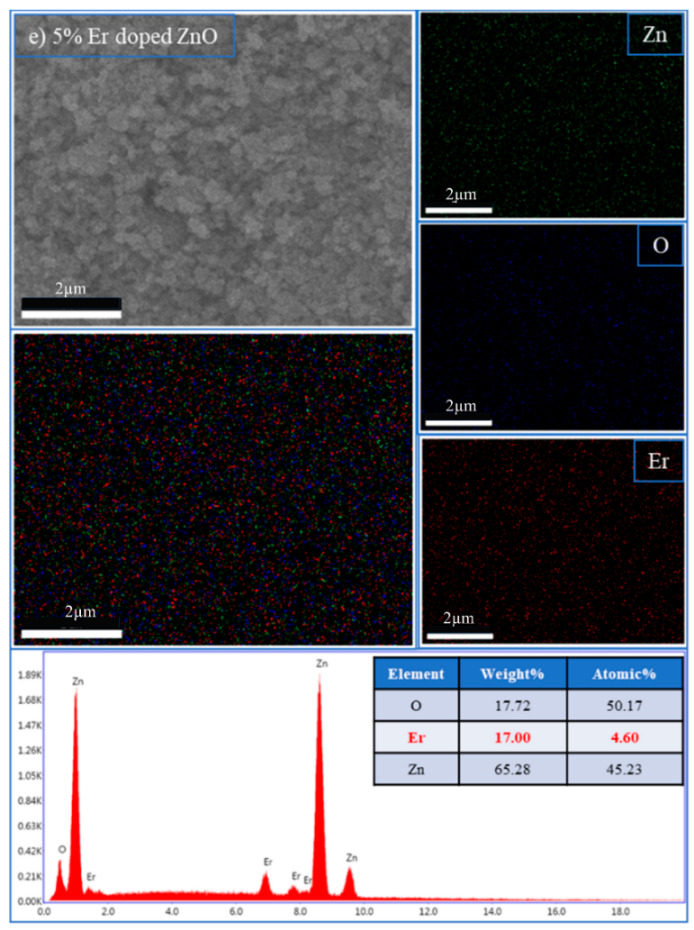

	
	
	
**Sample**	**Element**	**Weight%**	**Atomic%**
**0.1% Er:ZnO**	O_k_	19.22	49.35
	**Er_L_**	**0.32**	**0.08**
	Zn_k_	80.47	50.58
**0.5% Er:ZnO**	O_k_	16.36	44.64
	**Er_L_**	**1.20**	**0.31**
	Zn_k_	82.44	55.05
**1% Er:ZnO**	O_k_	14.35	41.25
	**Er_L_**	**3.52**	**0.97**
	Zn_k_	82.13	57.78
**2.5% Er:ZnO**	O_k_	13.87	41.17
	**Er_L_**	**8.41**	**2.38**
	Zn_k_	77.72	56.45
**5%Er:ZnO**	O_k_	17.72	50.17
	**Er_L_**	**17.00**	**4.60**
	Zn_k_	65.28	45.23

**Table A9:** Elemental composition and dopant distribution of Sm:ZnO thin films.

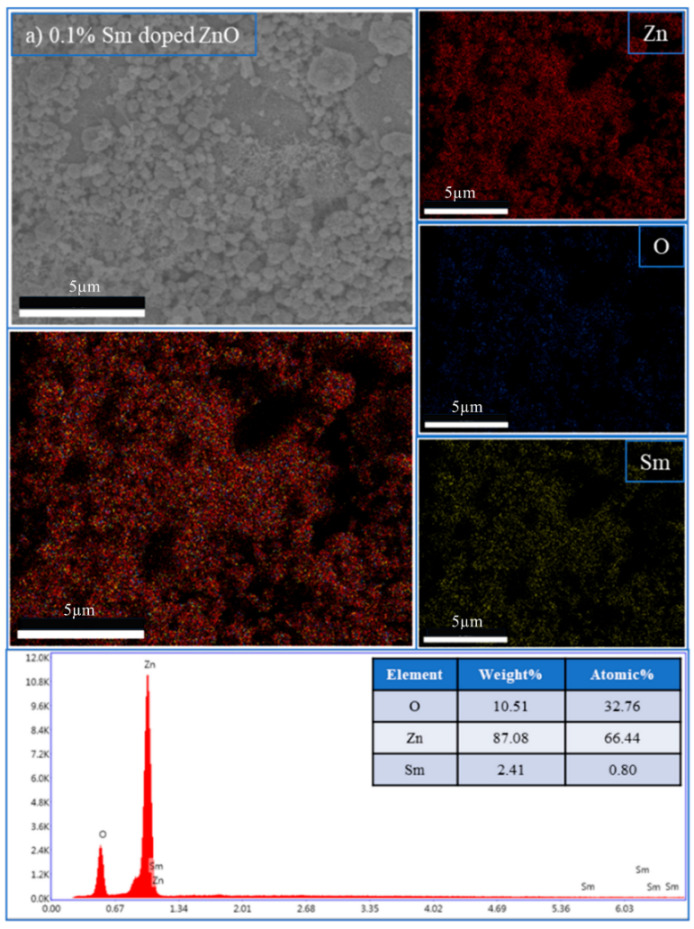
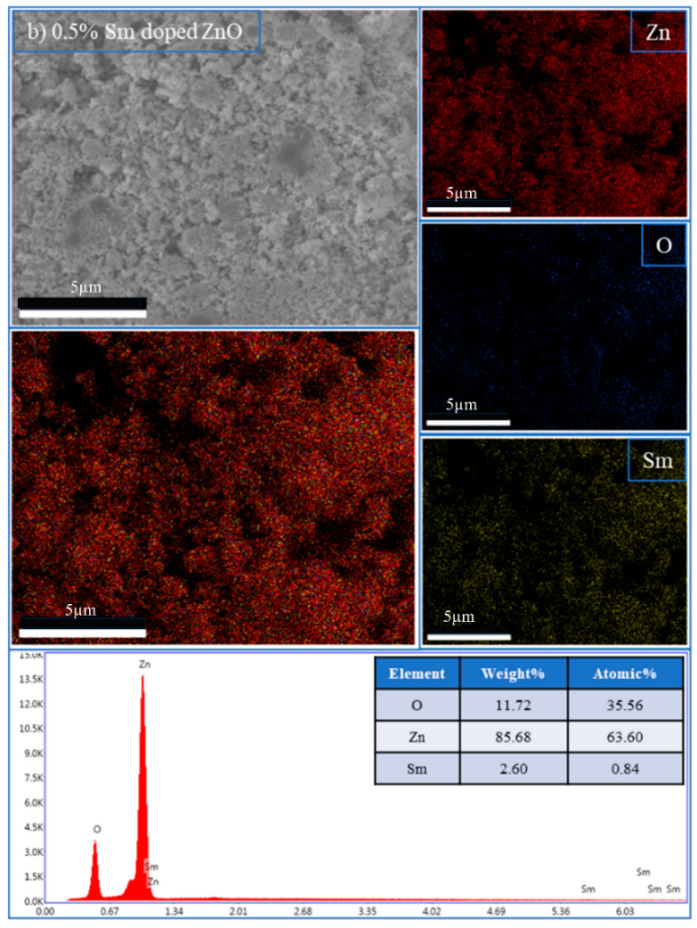

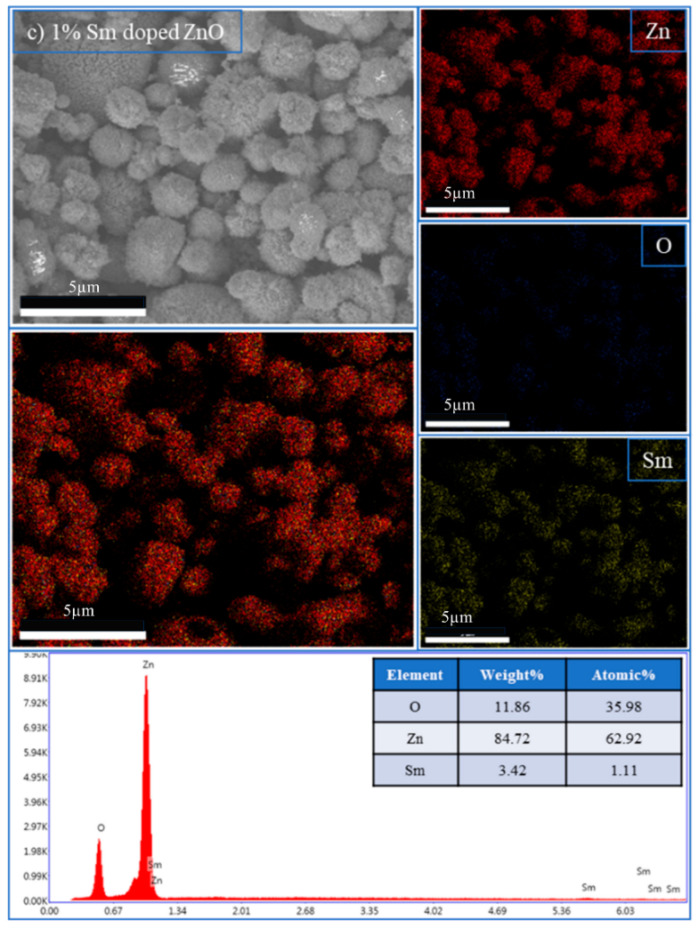
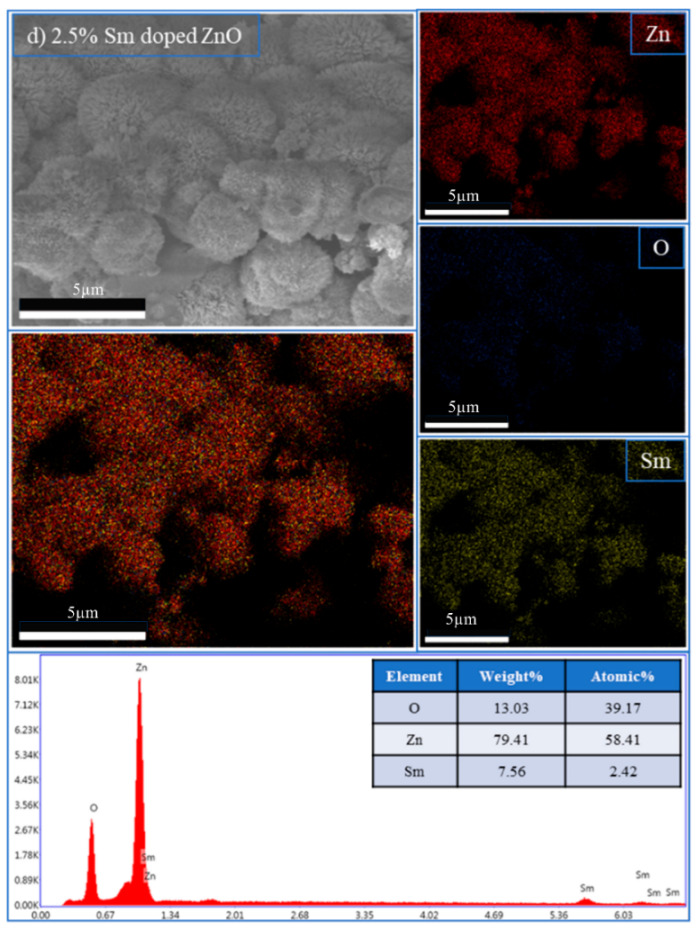

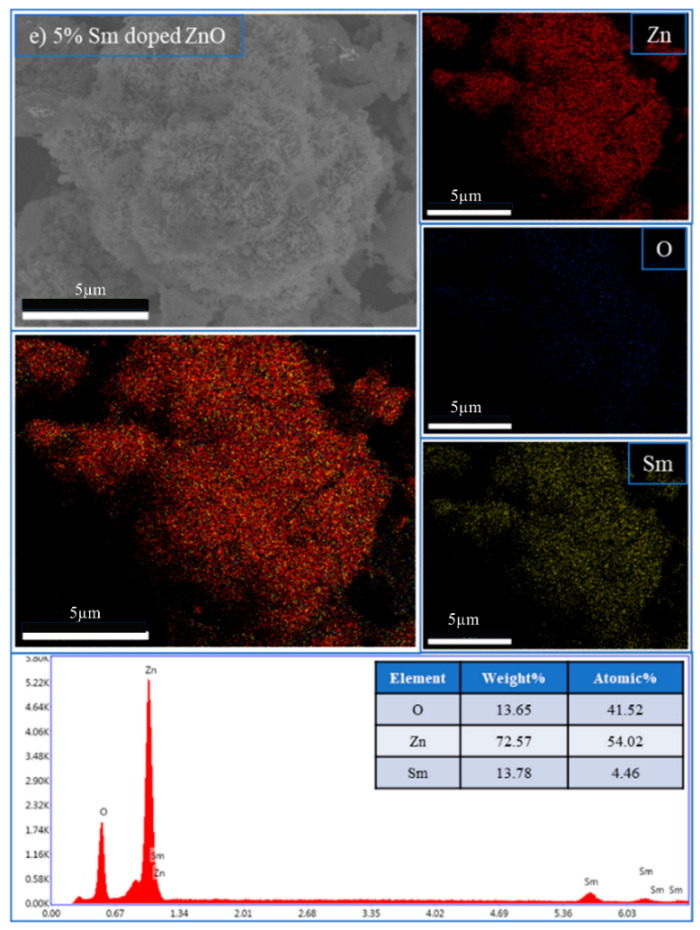

	
	
	
**Sample**	**Element**	**Weight%**	**Atomic%**
**0.1% Sm-doped ZnO**	O_k_	10.51	32.96
	**Sm_L_**	**2.41**	**0.60**
	Zn_k_	87.08	66.44
**0.5% Sm-doped ZnO**	O_k_	11.72	35.56
	**Sm_L_**	**2.60**	**0.84**
	Zn_k_	85.68	35.56
**1% Sm-doped ZnO**	O_k_	11.86	35.98
	**Sm_L_**	**3.42**	**1.11**
	Zn_k_	84.72	62.92
**2.5% Sm-doped ZnO**	O_k_	13.03	39.17
	**Sm_L_**	**7.56**	**2.42**
	Zn_k_	79.41	58.41
**5% Sm-doped ZnO**	O_k_	13.65	41.52
	**Sm_L_**	**13.78**	**4.46**
	Zn_k_	72.57	54.02

**Table A10:** XRD spectra of powders (blue lines) and obtained films on microscope slide glass from the respective powders (black lines).

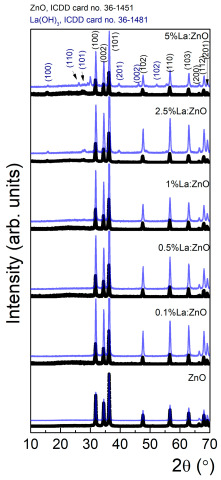
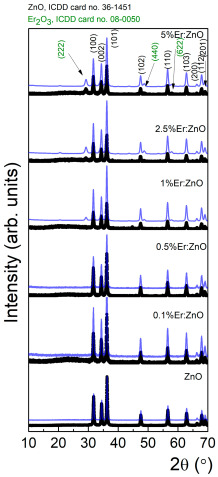
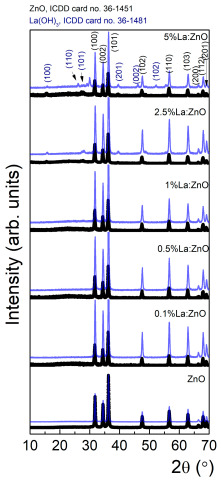

## Appendix B

Optical bandgap evaluation.

**Table A11:** Bandgap: **La:ZnO** series.

Samples	Eg (eV)
ZnO- Reference	3.341
**La:ZnO** (0.1%)	2.964
**La:ZnO** (0.5%)	2.947
**La:ZnO** (1%)	2.952
**La:ZnO** (2.5%)	2.958
**La:ZnO** (5%)	2.930

**Table A12:** Bandgap estimation using Tauc Plots for **La:ZnO** thin film series and optical absorbance spectra.

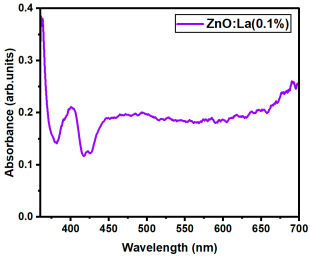
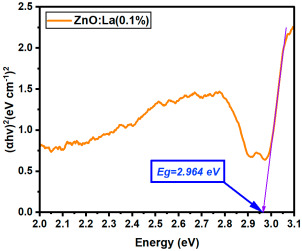

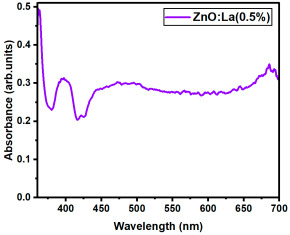
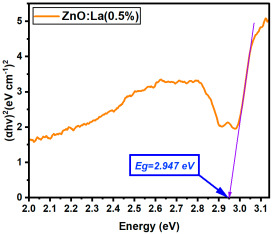

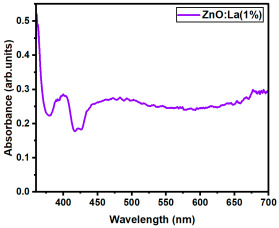
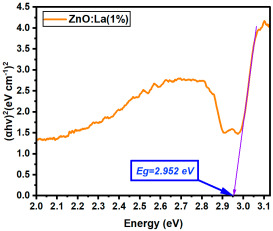

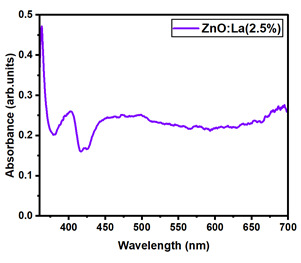
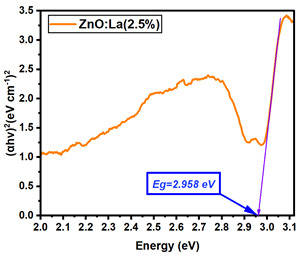

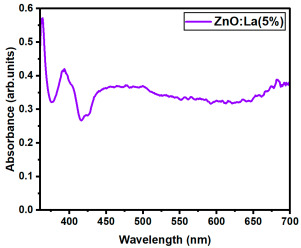
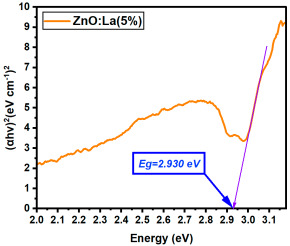

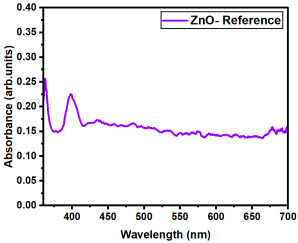
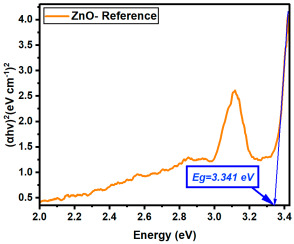

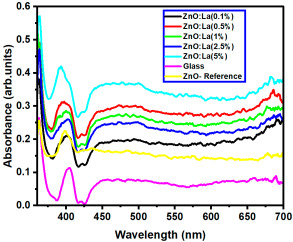

**Table A13:** Bandgap: **Er:ZnO** series.

Samples	Eg (eV)
ZnO- Reference	3.341
**Er:ZnO** (0.1%)	2.964
**Er:ZnO** (0.5%)	2.940
**Er:ZnO** (1%)	2.957
**Er:ZnO** (2.5%)	2.970
**Er:ZnO** (5%)	2.942

**Table A14:** Bandgap estimation using Tauc Plots for **Er:ZnO** thin film series and optical absorbance spectra.

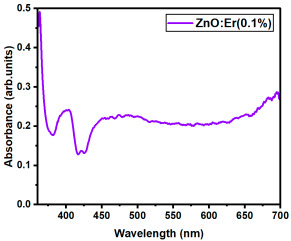
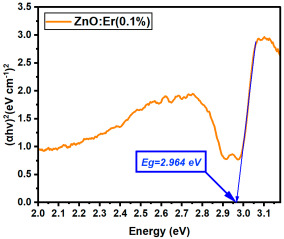

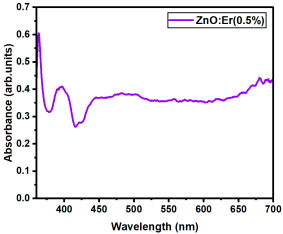
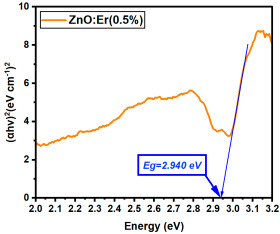

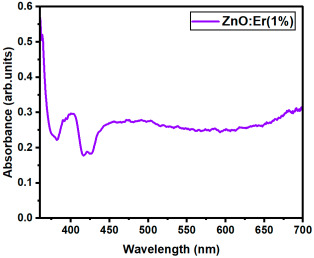
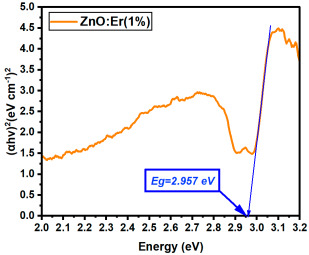

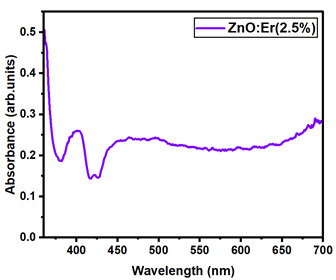
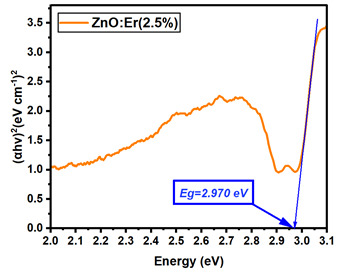

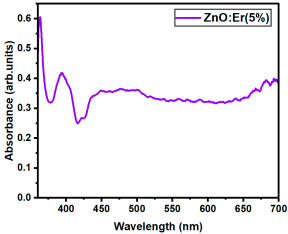
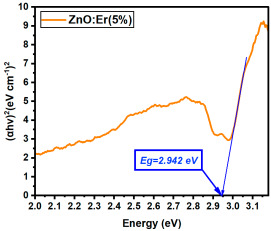

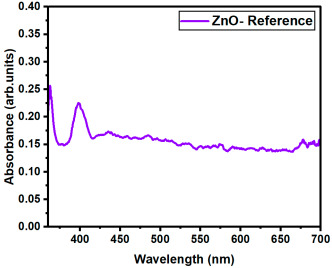
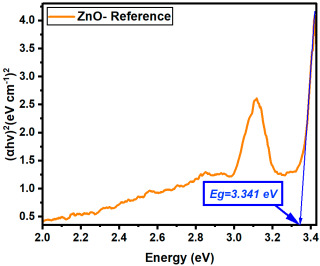

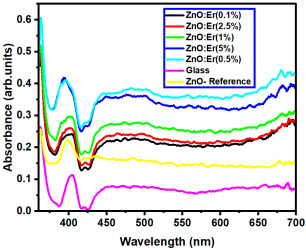

**Table A15:** Bandgap: **Sm:ZnO** series.

Samples	*Eg (eV)*
ZnO- Reference	3.341
**Sm:ZnO** (0.1%)	2.966
**Sm:ZnO** (0.5%)	2.967
**Sm:ZnO** (1%)	2.971
**Sm:ZnO** (2.5%)	2.965
**Sm:ZnO** (5%)	2.965

**Table A16:** Bandgap estimation using Tauc Plots for **Sm:ZnO** thin film series and optical absorbance spectra.
